# Heat in Wheat: Exploit Reverse Genetic Techniques to Discover New Alleles Within the *Triticum durum* sHsp26 Family

**DOI:** 10.3389/fpls.2018.01337

**Published:** 2018-09-19

**Authors:** Alessia Comastri, Michela Janni, James Simmonds, Cristobal Uauy, Domenico Pignone, Henry T. Nguyen, Nelson Marmiroli

**Affiliations:** ^1^Department of Chemistry, Life Sciences and Environmental Sustainability, University of Parma, Parma, Italy; ^2^Department of DiSBA, CNR, Institute of Bioscience and Bioresources, Bari, Italy; ^3^Department of DiTET, CNR, Institute of Materials for Electronics and Magnetism, Parma, Italy; ^4^John Innes Centre, Norwich Research Park, Norwich, United Kingdom; ^5^Division of Plant Sciences, University of Missouri, Columbia, MO, United States

**Keywords:** durum wheat, sHsp, TILLING, heat stress, KASP

## Abstract

Wheat breeding nowadays must address producers and consumers' desire. According to the last FAO report, a dramatic decrease in wheat production is expected in the next decades mainly due to the upcoming climate change. The identification of the processes which are triggered by heat stress and how thermotolerance develops in wheat is an active research topic. Genomic approach may help wheat breeding since it allows direct study on the genotype and relationship with the phenotype. Here the isolation and characterization of four members of the chloroplast-localized small heat shock proteins (sHSP) encoded by the *Hsp26* gene family is reported. Furthermore, two high throughput TILLING (Targeting Induced Local Lesions In Genomes) approaches *in vivo* and *in silico* were used for the identification of new alleles within this family. Small heat shock proteins are known to prevent the irreversible aggregation of misfolded proteins and contribute to the acquisition of thermotolerance. Chloroplast-localized sHSPs protect the photosynthetic machinery during episodes of high temperature stress. The modulation of the newly discovered genes within the sHsp26 family has been analyzed *in vivo* and by the ExpVIP platform widening the abiotic stress analysis; and their involvement in the heat stress response has been demonstrated. In addition, in this study a total of 50 TILLING mutant lines have been identified. A set of KASP (Kompetitive Allele Specific PCR) markers was also developed to follow the specific mutations in the ongoing backcrosses, applicable to high throughput genotyping approaches and usable in marker assisted selection breeding programs.

## Introduction

The next 50 years are expected to experience both a rise in mean temperatures and a decrease in annual precipitation across the Mediterranean Basin, the extent of which is likely to have a significant impact on agriculture (Field et al., 2012). An increasingly urgent priority for crop breeders has become, therefore, to improve the level of stress tolerance expressed in their working germplasm (Pereira, 2016). Wheat (*Triticum* spp.) supplies about 20% of the calories consumed by the world's population. Although most of the world's wheat production is focused on bread wheat (*T. aestivum*), around 5% is devoted to pasta wheat (*T. turgidum* var. *durum*) (IGC report 2014, http://www.thebioenergysite.com/reports/?category=33). The bulk of durum wheat production is concentrated in regions where terminal drought and high temperature stress are relatively common; the effect of these stresses is to constrain grain yield (Altenbach, 2012).

In response to high temperature stress, plants initially invoke their innate ability to survive (basal tolerance), a process which is later supported by acclimation, or the acquisition of tolerance. A commonly encountered stress response is an up-tick in the synthesis and the accumulation of heat shock proteins (HSPs), which are known to act as molecular chaperones. This abundant class of proteins support the maintenance of cellular homeostasis by contributing to the correct folding of nascent and stress-accumulated misfolded or denatured proteins (Vierling, 1991; Wang et al., 2004; Tyedmers et al., 2010) and protect specific transcription factors in cytosolic stress granules (McLoughlin et al., 2016). HSP synthesis and accumulation during high temperature stress has frequently been linked to the expression of thermotolerance (Marmiroli et al., 1989, 1994; Sun et al., 2012; Ni et al., 2018). Plant HSPs are classified, on the basis of their size, into five major groups, namely the HSP100s, HSP90s, HSP70s, HSP60s, and the small HSPs (sHSPs) (Al-Whaibi, 2011; Waters, 2013; Sharma et al., 2015). Of these, the latter are particularly abundant in plants exposed to high temperature stress; they are monomeric proteins ranging in size from 12–42 KDa (Waters, 2013) and are encoded by nuclear genes. Eleven sHSP subfamilies have been identified in the Angiosperms, of which six are deposited in the cytoplasm/nucleus; the other five localize to either the endoplasmic reticulum, the peroxisome, the chloroplast, or the mitochondria (Waters, 2013). Members of different subfamilies do not share a high degree of sequence similarity, although the overall secondary structure of the whole HSP protein family is relatively well conserved (Sarkar et al., 2009). A characteristic of all sHSPs is the presence of a central α-crystalline domain (ACD) (Bondino et al., 2012), within which lie two consensus regions (CRI and CRII), determining a β-sheet known to be essential for dimerization and higher order assembly (Poulain et al., 2010). The N terminal domain participates in substrate binding and binds denatured proteins (Basha et al., 2006; Jaya et al., 2009), while the C terminal domain is involved in the homo-oligomerization (Giese and Vierling, 2004) and the formation of high temperature stress granules (Kirschner et al., 2000). While almost every sHSP is induced by high temperature stress, some are induced exclusively either at a specific developmental stage(s) (Waters, 2013) or in response to other abiotic stress agents (Rampino et al., 2012). The involvement of a chloroplast small HSP in thermotolerance and thermomemory in Arabidopsis has been recently described (Zhong et al., 2013; Sedaghatmehr et al., 2016). The *Arabidopsis thaliana* genome encodes 19 sHSPs (Visioli et al., 1997; Scharf et al., 2001), compared to 23 in rice (Sarkar et al., 2009) and 36 in poplar (Waters et al., 2008). A number of putative sHSP-encoding genes have been isolated in wheat (Weng et al., 1991; Nguyen et al., 1993; Basha et al., 1999; Rampino et al., 2009, 2010, 2012; Pandey et al., 2015). Among these are genes encoding the chloroplast-localized HSP26 proteins, which are ubiquitous throughout the plant kingdom (Ul Haq et al., 2013). HSP26 interacts with photosystem II (Chauhan et al., 2011; Zhang et al., 2014; Hu et al., 2015), thought to be the most thermo-sensitive component of the photosynthetic machinery (Osteryoung and Vierling, 1994; Joshi et al., 1997). Because of their central role more efforts are needed to identify the complete gene sequences encoding for chloroplast sHSPs in *Triticum* species.

Here we describe the isolation and molecular characterization of the durum wheat family of *sHsp26* homologs, taking advantage of the available wheat genomic resources including the complete genome sequences and high quality gene models from the IWGSC CSS (Mayer et al., 2014), the Meracolous scaffolds (Chapman et al., 2015), the TGACv1 assembly (Clavijo et al., 2017), and the transcriptomic databases (Borrill et al., 2016). Moreover, we identified a collection of *de novo* alleles by following both an *in silico* and *in vivo* TILLING (Targeting Induced Local Lesions In Genomes) approaches (McCallum et al., 2000) using an exome-sequenced mutant populations (Krasileva et al., 2017) and a classical TILLING population (Uauy et al., 2009). The characterization of NILs starting from the mutant- identification to test the mutant lines for their thermo-tolerance and heat resilience is ongoing. To speed up the selection process and to develop a genomic tool for Marker Assisted Selection (MAS), we have developed a set of KASP (Kompetitive Allele Specific PCR) markers to follow the mutations through the generations.

## Materials and methods

### Plant materials and heat stress conditions

Two established ethyl methanesulfonate mutagenized populations of pasta wheat were analyzed, one based on cv. Kronos and the other on cv. Cham1 (Parry et al., 2009; Uauy et al., 2009). Grains (including those of both progenitor cultivars) were germinated in Petri dishes for 10 days at 5°C, then potted into soil and grown in a glasshouse under well-watered conditions.

For the heat stress treatment, durum wheat plants cv. Cham1 were grown in controlled chamber with the following conditions: light/dark 16/8 h with 25/20°C temperature for 10 days and then exposed to direct stress or acclimation. For the direct stress experiment, plants were heat stressed at 42°C for 2 h (S) and recovered for 2 h at 25°C (S+R). For the acclimation experiment, plants were acclimated by incubation at 34°C for 1 h (1 h) and 24 h (24 h) and subsequently exposed at 42°C for 2 h (24 h+S) and recovered for 2 h at 25°C (24 h+S+R). Control plants (Ctrl) were sampled immediately before the stress or the acclimation imposition. Four seedlings for each replication were sampled, pooled and immediately freezed with liquid nitrogen for RNA analysis.

### Identification of the *TaHsp26* sequence

The IWGSC CSS (International Wheat Genome Sequencing Consortium Chromosome Survey Sequencing) and the TGACv1 assembly were scanned from the Ensembl Plants database for wheat sequences matching that of *T. durum Hsp26.5* mRNA (AJ971373) using the BlastN algorithm. The genomic scaffolds of the TGACv1 assembly (Ensembl Plants release 35) carrying putative *Hsp26* genes were identified and then the BLAST tool was used for the *in silico* localization of *Hsp26* loci.

### DNA extraction, *TdHsp26* isolation and sequence analysis

Genomic DNA was extracted following Tai and Tanksley (1991) from a 50 mg sample of 11 days old seedling leaves harvested from individual M_2_ plants of both the cv. Cham1 and cv. Kronos populations. The full *TdHsp26-A1, -A2, -B1* sequences and the partial –*A3* sequences were PCR-amplified from both cv. Cham1 and cv. Kronos template using GoTaq Long PCR 2X Master Mix (Promega, Madison, WI, USA) in 30 μL reactions each containing 10–20 ng template and 0.4 μM of each primer. The primers, designed using Primer3 v4.0 (bioinfo.ut.ee/primer3-0.4.0/, Untergasser et al., 2012) on the basis of archival sequence, were A1-9F/A1-4R for *TdHsp26-A1*, A2-24F/A2-18R for *-A2*, A3-20F/A3-29R for *-A3* and B1-5F/R for *-B1* (sequences given in Table 1). The reactions were initially denatured (94°C/2 min), then subjected to 40 cycles of 94°C/15 s, 60°C/30 s, 72°C/90 s, and completed by a final extension of 72°C/10 min. The amplicons were electrophoretically separated through a TAE agarose gel, extracted from the gel using a NZYGelpure kit (NZYtech, Lisbon, Portugal) and submitted for sequencing (Eurofins Genomics, Edersberg, Germany). The chromosomal origin of each amplicon was confirmed by aneuploid analysis, based on the cv. Chinese Spring set of nulli-tetrasomic and ditelosomic lines. Primers directed at the *T. aestivum Actin* gene (AB181991) (Rocchi et al., 2012) were used as a positive PCR control. The isolated gene sequences were aligned and analyzed. In addition, of all *TdHsp26* genes the promoter sequences of 1,400 bp, recovered by the TGAC assembly, were searched for the presence of cis-active elements by using the PlantCARE database.

**Table 1:** Primer sequences used for gene isolation, chromosomal localization, RT qPCR analysis, and TILLING.

**Primers pairs**		**Sequence 5**^**′**^−3^**′**^		**Target and application**	**Amplicon size on gene sequence (bp)**
**Forward**	**Reverse**	**Forward**	**Reverse**	
A1–9F	A1–4R	TGTTGGGCCTCCTGATCG	AGCCTCAGATGCAGGGTAC	*TdHsp26-A1* isolation and chromosomal localization	1,171
A2–24F	A2–18R	CCACCAGACAATCACTGCAA	CAGGGTACAGTCTCACACG	*TdHsp26-A2* isolation and chromosomal localization	939
A3–20F	A3–29R	GGCGAAGATCTGCCAAAGTAT	AACCAGCACAACCCTCTA	*TdHsp26-A3* isolation and chromosomal localization	1,106
B1–5F	B1–6R	GACACTCTCTCGTTTCAATTCTC	GTTATCAGCTTCTTCCGGG	*TdHsp26-B1* isolation	1,182
B1–17F	B1–6R	TCTCCAACCAGGTACGCC	GTTATCAGCTTCTTCCGGG	*TdHsp26-B1* 1st round HRM	1,378
B1–PT10F	B1–PT10R	CGATGCGGCAGATGCTT	TGACGAGCGCGTCGC	*TdHsp26-B1* 2nd round HRM, chromosomal localization and RT qPCR analysis	211
A1–PT31F	A1–PT31R	CCAGGCCCAGAACGCT	CCTCCTTcTCGTCCTCCATa	RT qPCR	338
A2–PT24F	A2–PT25R	CGCTaTaGTCAGCCGCCTt	GCGaCGCTGGACTGCa	RT qPCR	206
A3–PT27F	A3–PT27R	ATGGCTGcaGCGAACGCt	CGTCGACGGAGTTGTCCCTa	RT qPCR	172
ACT–Fw	ACT–Rev	TCCTGTGTTGCTGACTGAGG	GGTCCAAACGAAGGATAGCA	*Actin* housekeeping	240

Sequence analysis, multiple alignment, Neighbour Joining tree and were performed by using DNAMAN software (Lynnon Biosoft, Quebec, Canada).

### *In vitro* and *in silico TdHsp26* transcription profiling

The RNA from control and heat treated seedlings of the cv. Cham1 was extracted with RNeasy Plant Mini Kit (Qiagen, Hilden, Germany),cDNA was generated with QuantiTect Rev.Transcription Kit (Qiagen). 20 ng/μL cDNA were used for Real Time (RT) qPCR analysis by using the ABI PRISM® 7000 Sequence Detection System (Applied Biosystems, Foster City, USA). The reactions were set up in 10 μL reaction volume with 2X SybrGreen Master Mix (Life Technologies, Carlsbad, CA, USA) and 25 nM of gene-copy specific forward and reverse primers A1-PT31F/A1-PT31R, A2-PT24F/A2-PT25R, A3-PT27F/A3-PT27R, B1-PT10F/B1-PT10R (Table 1); manufacturer indications were followed for the cycling conditions. Transcription of *TaActin* gene (AB181991) was used for normalization, following the conditions indicated in Rocchi et al. (2012). Three technical replicates were made for each Real Time qPCR analysis, the relative quantitation (RQ) analysis was determined by using the 2^−ΔΔ*Ct*^ method (Livak and Schmittgen, 2001) and shown in chart as log2(RQ).

The *in silico* ExpVIP platform (Wheat Expression Browser, www.wheat-expression.com) was also queried and the transcriptional profile of the *TdHsp26* genes in response to abiotic stresses on the basis of RNAseq data was analyzed. Transcript abundances are expressed in log_2_ (tpm), (transcript per million; see Borrill et al., 2016 for details). In this paper only the data in response high temperature and/or drought (Liu et al., 2015) were considered.

### TILLING for the detection of *de novo TdHsp26* alleles

A Cham EMS population composed by a total of 960 samples was used in this experiment. In the TILLING assays, the heteroduplexes are usually derived from the melting and re-annealing of wild type and mutant amplicons, generated by 2-fold pooling of genomic samples before PCR as also reported in Botticella et al. (2011).

The genotypes were randomly selected within the mutant population and DNA were pooled with a 2-fold approach.For this first round of amplification, *-B1* gene specific primers targeting the 3'- and 5'- UTR of cv. Cham1 *TdHsp26-B1* (B1-17F/B1-6R, Table 1) were used for the first PCR, generating a 1,378 bp B1 specific amplicon. The second step of the mutation detection was carried with HRM by using internally positioned specific primer pair B1-PT10F/R (Table 1) to produce a 211 bp amplicon, which included the functional methionine-rich domain (MrD) and the ACD. The presence of the mutation has been detected comparing the ΔF/T curves (**Figure 2D**), produced by the HRM software, the observation of dF/dT curves The specificity of the B1-PT10F/R primer pair was validated by aneuploid analysis (Supplementary Figure S1).

The first round was conducted in 12.5 μL PCR contained 10–20 ng template, GoTaq Hot Start Colorless 2X Master Mix (Promega) and 0.25 μM of each primer, applying the same cycling conditions described above for the *TdHsp26* isolation experiment. 1 μL of a 1:60 dilution of the resulting amplicon was used as template for the second PCR reaction (10 μL final volume), run on a ABI PRISM® 7900 HT device (Applied Biosystems), containing MeltDoctor^TM^ HRM 2X Master Mix (Life Technologies, Carlsbad, CA, USA) and 0.3 μM of the primer pair B1-PT10F/R (Table 1). High Resolution Melting (HRM) was used to detect *sHsp26* mutants (Botticella et al., 2011). After the amplification the melting step was performed by using SDS software v2.4 (Applied Biosystems), the reaction mixtures were denatured (95°C/10 min), cycled 40 times through 95°C/15 s, 60°C/60 s, then melted (95°C/10 s, 60°C/60 s, 95°C/15 s, 60°C/15 min). The resulting melting curves were analyzed using HRM software v2.0.1 (Applied Biosystems) following a manual setting of the pre-melt and post-melt regions. The positive pools putatively containing a mutant DNA were sequenced to verify the presence of *TdHsp26* mutations. Additional mutations to *TdHsp26-A1* and *B1* in the cv. Kronos population were identified from http://www.wheat-tilling.com using IWGSC CSS transcript information Traes_4AS_8BA1E69CA for -*A1*, and Traes_4BL_3C1C91A9C for *-B1*.

### KASP markers generation and analysis

The KASP assay is a SNP genotyping platform that becomes routinely used in marker assisted selection for plant breeding (Ertiro et al., 2015) and also to validate a selection of the *in silico*-detected SNPs (Trick et al., 2012; King et al., 2015). Briefly, from 11 to 16 plants from each of 13 putative mutant lines were sampled to confirm the presence of the mutation and to determine its zygosity; two technical replicates were run for each plant. The primers used for these assays were designed using the PolyMarker system (polymarker.tgac.ac.uk, Ramirez-Gonzalez et al., 2015) where possible. In the absence of the necessary data (e.g., for chromosome 4B), the primers were manually designed. The primers carried either a FAM (5'-GAAGGTGACCAAGTTCATGCT) or a HEX (5′-GAAGGTCGGAGTCAACGGATT) compatible tail, with the variant nucleotide lying at the 3′ end (Supplementary Table S1). The primer mix, as recommended by LGC Genomics, comprised 46 μL dH_2_O, 30 μL 100 μM common primer and 12 μL of each tailed primer (100 μM). Each assay involved 10–20 ng template, 2 μL 2X KASP Master mix (LGC Genomics) and 0.07 μL primer mix. The PCR protocol comprised a 94°C/15 min denaturation, followed by 10 touchdown cycles of 94°C/20 s, 65°C [reducing by 1°C per cycle]/60 s, and 30 cycles of 94°C/20 s, 57°C/60 s. The plates were read using a Tecan Safire plate reader **(**Tecan Group Ltd. Männedorf, Switzerland) at room temperature. Data analysis was performed manually using Klustercaller v2.22.0.5 software (LGC Genomics). The different clusters representative of the wild type and mutated *TdHsp26*, resulted by the KASP markers analyses (Semagn et al., 2014) on the parental and putative mutant lines for *TdHsp26*, were compared. The zygosity of the mutant plants was assigned accordingly (heterozygous or homozygous).

### Bioinformatic analysis

The BLAST-enabled search for *TdHsp26.5* (AJ971373) sequence used the following databases: IWGSC/URGI (urgi.versailles.inra.fr/blast/?dbgroup=wheat_survey&program = blastn); EnsemblPlants *Triticum aestivum* TGACv1 Assembly and IWGSC1+popseq (http://plants.ensembl.org/Triticum_aestivum/Tools/Blast?db=core); WGS w7984_Meraculous Scaffolds (http://www.cerealsdb.uk.net/cerealgenomics/CerealsDB/blast_WGS.php); NCBI (blast.ncbi.nlm.nih.gov/Blast.cgi) and TREP (wheat.pw.usda.gov/ITMI/Repeats/; Wicker et al., 2002). Sequence analysis, assembly, multiple alignment and the homology calculation were performed using routines implemented in DNAMAN (Lynnon Biosoft). FGENESH and FSPLICE (Solovyev et al., 2006) analysis were used to determine gene structure; protein structure alignments were determined using the PROMALS3D program (prodata.swmed.edu/promals3d/promals3d.php; Pei et al., 2008). Protein localization relied on ChloroP 1.1 (www.cbs.dtu.dk/services/ChloroP/).

The cv. Kronos derived mutants were retrieved from the wheat-tilling.com and their putative effect on functionality was predicted by PARSESNP (blocks.fhcrc.org/proweb/parsesnp/, Taylor and Greene, 2003). The design of the TILLING primers was based on CODDLE software (blocks.fhcrc.org/proweb/input/). The promoters have been analyzed with the PlantCARE database (http://bioinformatics.psb.ugent.be/webtools/plantcare/html/).

## Data availability

The isolated sequences have been deposited and are available at the EMBL Nucleotide Sequence Database. The specific accession numbers are reported in **Table 4**. Supplementary Figure S1 shows the chromosome localization of the TdHsp26 genes. Supplementary Figure S2 contains the multiple alignment of the sHsp26 gene sequences characterized in the durum wheat Cv. Cham1. Supplementary Figure S3 contains the multiple alignment of the sHSP26 protein sequences with the predicted structural features. The full nucleotide sequences of the oligoes used in this work, including the KASP markers, are reported in Table 1 and Supplementary Table S1. The TILLING lines of Cv. Kronos TILLING population listed in Supplementary Tables S2, S3 are available upon request at www.seedstor.ac.uk. The mutant lines of the Cv. Cham1 are listed in Supplementary Table S4. Supplementary Table S5 contains the expression analysis data of the direct heat stress and acclimation real time experiments and the *in silico* trascriptomic data recovered by ExpVIP.

## Results

### Identification and genomic characterization of the *TdHsp26* gene family

**Figure 1:**
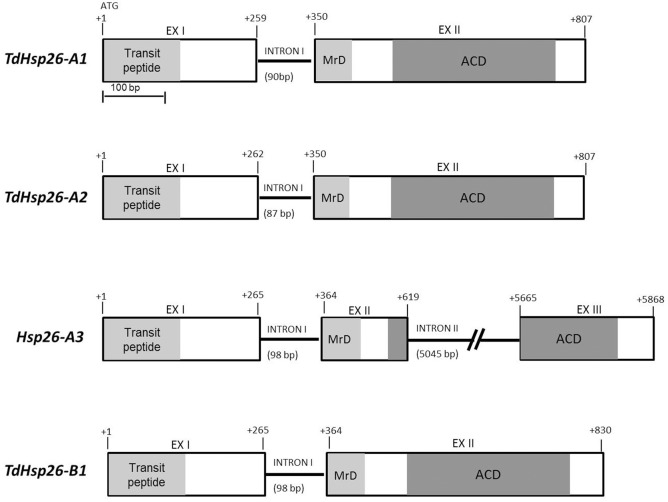
*TdHsp*26 gene structures. The conserved N-terminal MrD (Methionine-rich Domain) amphipathic α-helix is highlighted in light gray and the ACD in dark gray. The exon/intron junctions are indicated.

*TdHsp26.5* (AJ971373) matching sequences in either of the two sub-genomes A and B were considered, provided that their level of nucleotide identity was >89.2% and their E-value was <5.5E−10. The matches were grouped on the basis of chromosome position, scaffold and transcript annotation (Table 2) from which the complete putative *TaHsp26* gene sequences were reconstructed. The process led to recognize four putative *TaHsp26* genes, namely *TaHsp26-A1, -A2, -A3*, and *-B1*. The three A genome loci all mapped on the short arm of chromosome 4A, while the single B genome locus mapped on 4BL (Table 2) with *TaHsp26-A2* and *-A3* matching the same TGACv1_scaffold_307094 (Table 2). The analysis of the WGS Meraculous Scaffolds revealed that *TaHsp26-A1, -A2*, and -*A3* mapped on scaffolds 4146766, 2124046, and 731871, respectively that belong to the same single bin (chromosome arm 4AS, position 57.601 cM; Mascher et al., 2013), suggesting that these genes are in tandem on the 4AS chromosome. The presence of a potential *sHsp26* cluster is reminiscent of the situation in tomato, where *sHsp*s of variable feature, class and function lie close to one another (Goyal et al., 2012). The successful design of locus-specific primer pairs (Table 1) confirmed the presence of four gene copies in cv. Cham1: *TdHsp26-A1Ch* (LT220905)*, -A2Ch* (LT220907)*, -A3Ch* (LT220909), and *-B1Ch* (LT220911). The gene's topography is shown in Figure 1: two exons and one intron for *TdHsp26-A1Ch, -A2Ch*, and -*B1Ch* and two introns for the *-A3Ch* gene. The second intron of the *-A3Ch* gene is approximately 5602 bp, so far only a partial gene sequence was identified for the *-A3Ch* (LT220909. The *-A3* gene structure and its complete coding sequence (CDS) was deduced from the TGACv1 assembly (so far named *Hsp26-A3*, Supplementary Figure S2). The CDS of -*A1Ch, -A2Ch, -A3*, and -*B1Ch* genes are 717, 720, 723, and 732 bp respectively (Supplementary Figure S2). The chromosome arm locations were further confirmed by amplifying template prepared from the homoeologous group 4 aneuploids of cv. Chinese Spring (Supplementary Figure S1).

**Table 2:** *In silico* analysis of the EnsemblPlants database and the strategy used to isolate the *TdHsp26* gene family.

Assembled Ta^*^gene	**Isolated Td∧gene/cDNA**	**Chr**	**TGACv1 genomic location**	**Orientation**	**Transcript ID with TGAC annotation**	**Transcript ID with IWGSC CSS annotation**	**Query start**	**Query end**	**%ID**	***E*-value**
TaHsp26-A1	TdHsp26-A1Ch	4AS	TGACv1_scaffold_307667_4 AS:25170-25429	+	TRIAE_CS42_4AS_TGACv1 _307667_AA1022530	Traes_4AS_8BA1E69CA	1	260	99.6	1.1E−140
			TGACv1_scaffold_307667_4 AS:25518-25976	+	TRIAE_CS42_4AS_TGACv1 _307667_AA1022530	Traes_4AS_8BA1E69CA	259	717	99.8	0.0
TaHsp26-A2	TdHsp26-A2Ch	4 AS	TGACv1_scaffold_307094_4 AS:65967-66042	–	TRIAE_CS42_4AS_TGACv1 _307094_AA1016920	Traes_4 AS_2272D0413	29	104	90.8	1.5E−16
			TGACv1_scaffold_307094_4 AS:65891-65966	–	TRIAE_CS42_4 AS_TGACv1 _307094_AA1016920	Traes_4AS_2272D0413	102	177	96.1	4.3E−26
			TGACv1_scaffold_307094_4 A S:65811-65875	–	TRIAE_CS42_4 AS_TGACv1 _307094_AA1016920	Traes_4AS_2272D0413	196	260	89.2	5.5E−10
			TGACv1_scaffold_307094_4 AS:65267-65725	–	TRIAE_CS42_4 AS_TGACv1 _307094_AA1016920	Traes_4AS_2272D0413	259	717	97.2	0.0
TaHsp26-A3	TdHsp26-A3Ch	4AS	TGACv1_scaffold_307094_4 AS:26523-26598	–	TRIAE_CS42_4AS_TGACv1 _307094_AA1016910	Traes_4 AS_049E43B8B	29	104	93.4	2.5E−21
			TGACv1_scaffold_307094_4 AS:26364-26522	–	TRIAE_CS42_4 AS_TGACv1 _307094_AA1016910	Traes_4AS_049E43B8B	102	260	96.2	1.8E−68
			TGACv1_scaffold_307094_4 AS:26013-26267	–	TRIAE_CS42_4 AS_TGACv1 _307094_AA1016910	Traes_4AS_049E43B8B	259	513	98.4	1.6E−130
			TGACv1_scaffold_307094_4 AS:20762-20970	–	TRIAE_CS42_4 AS_TGACv1 _307094_AA1016910	Traes_4AS_049E43B8B	509	717	97.1	2.6E−98
TaHsp26-B1	TdHsp26-B1Ch	4BL	TGACv1_scaffold_320317_4 BL:105036-105111	+	TRIAE_CS42_4 BL_TGACv1 _320317_AA1035010	Traes_4 BL_3C1C91A9C	29	104	90.8	1.5E−16
			TGACv1_scaffold_320317_4 BL:105112-105270	+	TRIAE_CS42_4BL_TGACv1 _320317_AA1035010	Traes_4 BL_3C1C91A9C	102	260	97.5	3.1E−73
			TGACv1_scaffold_320317_4 BL:105367-105652	+	TRIAE_CS42_4BL_TGACv1 _320317_AA1035010	Traes_4 BL_3C1C91A9C	259	544	96.2	2.6E−132
			TGACv1_scaffold_320317_4 BL:105658-105834	+	TRIAE_CS42_4BL_TGACv1 _320317_AA1035010	Traes_4 BL_3C1C91A9C	541	717	97.7	5.6E−84
NM			TGACv1_scaffold_307094_4 AS:36607-36698	–	NM	n/a	626	717	97.8	4.9E−38
NM			TGACv1_scaffold_307094_4 AS:35725-35808	–	NM	n/a	634	717	98.8	1.2E−35

*The TdHsp26.5 mRNA sequence (AJ971373) was used as the query sequence. The TGACv1 matches indicate the location of the hit on the scaffold, the transcript ID indicates which genes are present at the genomic site according to EnsemblPlants. The corresponding transcript ID in the IWGSC CSS annotation is also reported. Chr: Chromosomal position. n/a: not available. NM indicates “No Matches” with annotated genes*.

* *Ta, Triticum aestivum; ^∧^Td, Triticum durum*.

With the exception of the long intron II retrieved in *Hsp26-A3*, only minor variations in the exon and intron lengths were observed among the other sequences (Table 3, Figure 1). The sequence comparison of the *-A1, -A2, -A3*, and *-B1* CDS (Supplementary Figure S2) with known *Triticum* spp. s*Hsp26* sequences surveyed in Table 4 showed that -*A1Ch* shared 99.72% sequence identity with *TdHsp26.5* (AJ971373)*, -A2Ch* 99.44% identity with the *T. dicoccoides sHsp TdiHSP26.4* (AJ971375), *-A3* shared 99% with both the hsp26.6-i allele (AF097658, Campbell, 1998) and the Tahsp26.6 (X58280) reported for the cv. Mustang in *T. aestivum* (Weng et al., 1991). The *-B1Ch* CDS showed 100% identity with *TaHsp26-g* (AF097657) described by Campbell (1998).

**Table 3:** *TdHsp26-A1Ch, -A2Ch, Hsp26-A3* and *-B1Ch* gene structures and predicted protein products.

**Sequence**	**Exon I (bp)**	**Exon II (bp)**	**Exon III (bp)**	**Intron I length (bp)**	**Intron II length (bp)**	Gene sequence length (bp)^*^	**Protein size (Amino Acid)^a^**	**Protein weight (KDa)^a^**
*TdHsp26-A1Ch*	1–259	350–807	–	90	–	807	238	26.528
*TdHsp26-A2Ch*	1–262	350–807	–	87	–	807	239	26.400
*Hsp26-A3*	1–265	364–619	~5665–5868	98	~5045	~5868	240	26.680
*TdHsp26-B1Ch*	1–265	364–830	–	98	–	830	243	26.826

* *Including Introns*.

a *The size and weight of the translation products were predicted with DNAMAN software*.

**Table 4:** Survey of the annotated *Triticum* spp. *sHsp26* sequences and references.

**Gene**	**Allele ID**	**Protein ID**	**Mw (KDa)**	**Organism**	**Wheat Cv**	**References**
*Hsp26.5*	AJ971373	CAI96515	26.489	*T. turgidum* subsp. *durum*	Creso	Rampino et al., 2009
*Hsp26.6g*	AF097657	AAC96315	26.844	*T. aestivum*	Mustang	Campbell, 1998
*Hsp26.6e*	AF097656	AAC96314	26.469	*T. aestivum*	Mustang	Campbell, 1998
*Hsp26.6i*	AF097658	AAC96316	26.583	*T. aestivum*	Mustang	Campbell, 1998
*Hsp26.6m*	AF097659	AAC96317	26.560	*T. aestivum*	Mustang	Campbell, 1998
*Hsp26.6*	X58280	CAA41219	26.566	*T. aestivum*	Mustang	Weng et al., 1991
*sHsp*	HM802264	ADN97108	26.844	*T. aestivum*	C306	Kumar et al., unpublished
*Hsp26.6B*	X67328	CAA47745	26.557	*T. aestivum*	Mustang	Nguyen et al., 1993
*Hsp26.4*	AJ971370	CAI96512	26.417	*T. turgidum* subsp. *dicoccoides*	MG29896/212	Rampino et al., unpublished
*Hsp26.8*	AJ971372	CAI96514	26.734	*T. turgidum* subsp. *dicoccum*	MG5473/295	Rampino et al., unpublished
*Hsp26.6*	AJ971371	CAI96513	26.554	*T. turgidum* subsp. *dicoccoides*	MG29896/212	Rampino et al., unpublished
*Hsp26.6*	AJ971374	CAI96516	26.564	*T. monococcum*	ID362	Rampino et al., unpublished
*Hsp26.5*	AJ971375	CAI96517	26.547	*T. monococcum*	ID529	Rampino et al., unpublished
*TdHsp26-A1Ch*	LT220905	CZQ76680	26.528	*T. turgidum* subsp. *durum*	Cham1	This paper
*TdHsp26-A1Kr*	LT220906	CZQ76681	26.489	*T. turgidum* subsp. *durum*	Kronos	This paper
*TdHsp26-A2Ch*	LT220907	CZQ76682	26.400	*T. turgidum* subsp. *durum*	Cham1	This paper
*TdHsp26-A2Kr*	LT220908	CZQ76683	26.417	*T. turgidum* subsp. *durum*	Kronos	This paper
*TdHsp26-A3Ch*	LT220909	CZQ76684		*T. turgidum* subsp. *durum*	Cham1	This paper
*TdHsp26-A3Kr*	LT220910	CZQ76685		*T. turgidum* subsp. *durum*	Kronos	This paper
*TdHsp26-B1Ch*	LT220911	CZQ76686	26.826	*T. turgidum* subsp. *durum*	Cham1	This Paper

The presence for almost all the previously annotated *Hsp* genes, of the mRNA sequences rather than the complete genomic sequence, does not allow any speculation regarding the presence of any pseudogene.

### Analysis of the predicted *TdHsp26* polypeptides

**Figure 2:**
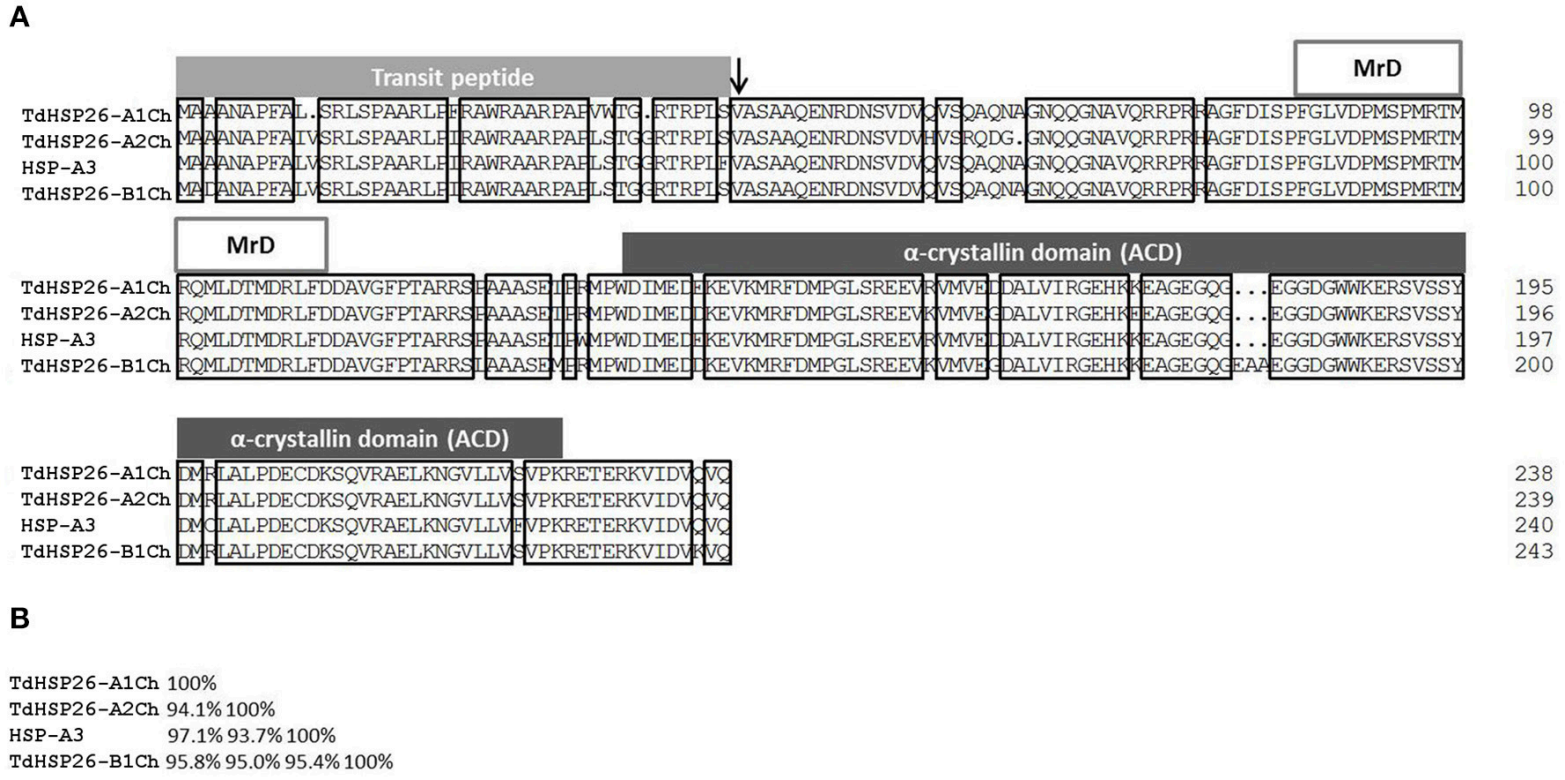
The TdHSP26-A1Ch, -A2Ch, -B1Ch and the predicted HSP26-A3 proteins. **(A)** Alignment of the deduced protein sequences, showing a schematic representation of their structure. The transit peptide cleavage site is arrowed. **(B)** Homology between the four TdHSP26 proteins. MrD, Methionine-rich Domain.

The retrieved *Hsp*26 sequences were analyzed for their predicted protein sequences. All proteins possess a typical sHSP26 topology characterized by the presence of the transit peptide that should direct the mature protein into the chloroplast, the N-terminal domain containing the conserved Methionine-rich Domain amphipathic α-helix (MrD) and the ACD domain that consists of the Conserved Region I (CRI), the Conserved Region II (CRII) and the β6 domain the N terminal MrD amphipathic α-helix, the ACD CRI and CRII, and the β6 domain (Scharf et al., 2001; van Montfort et al., 2001; Bondino et al., 2012; Waters, 2013; Figure 2A). The genes encoded for proteins of 238 (TdHSP26-A1Ch: CZQ76680), 239 (-A2Ch: CZQ76682), 240 (HSP26-A3), and 243 (-B1Ch: CZQ76686) residues. Pair-wise comparisons showed that the four proteins shared 94.1–97.1% sequence identity (Figure 2B).

When the comparison was extended to some of the surveyed chloroplast-localizing sHSP isolated in other plant species, it was clear that their MrD and the ACD CRI and CRII were shared with all the other chloroplast-localizing proteins (Supplementary Figure S3). Inspecting the variability among the sHSP26 sequences retrieved in *Triticum* is important to gain new insights about this multigenic family in polyploid species. As expected, *Hsp26* genes and alleles retrieved in *T. aestivum* and *T. durum* were highly similar in particular in the N terminal domain, which typically is the most variable part of the protein (Waters and Vierling, 1999; Bondino et al., 2012; Waters, 2013). The most prominent difference between TdHSP26-A1Ch and -A2Ch occurred within the N terminal domain: the sequence of residues 60–64 in -A1Ch was QAQNA (the same for 62-66 in –A3 and in -B1Ch), while that of 62–66 in -A2Ch was RQDG- (Figure 2A, Supplementary Figure S3). This difference may influence substrate binding affinity, thereby conferring a difference in the specificity of the sHSP26 isoforms. In TdHSP26-B1Ch, the tripeptide EAA has been inserted between the ACD CRI and CRII, as noted also by Campbell (1998); this arrangement may produce a more flexible tertiary structure (Figure 2A, Supplementary Figure S3). The sequence comparison showed that TdHSP26-A1Ch shared 99.2% identity with TdHSP26.5 (CAI96515), the difference being due to one substitution in the N terminal domain and another in the ACD. When compared to the TaHSP26.6-m (AAC96317, Campbell, 1998) sequence present in bread wheat cv. Mustang, the level of identity with TdHSP26-A1Ch was 99.6%, while it was 97.9% between -A1Ch and TaHSP26.6B (CAA47745), a second product harbored by cv. Mustang (Nguyen et al., 1993); the likelihood is that TaHSP26.6B and TaHSP26.6-m are encoded by different alleles. The TdHSP26-B1Ch sequence was identical to that of both of the bread wheat proteins TasHSP (ADN97108) and TaHSP26.6g (AAC96315) (Campbell, 1998). TdHSP26-A2Ch shared 99.6% identity with the *T. dicoccoides* protein HSP26.4 (CAI96512) and 100% identity with the cv. Chinese Spring sequence Traes 4AS_2272D0413. HSP26-A3 shared 98% identity with the *T. aestivum* HSP26.6 *(*TaHSP26.6i; AAC96316) encoded by the hsp26.6-i allele (AF097658) (Campbell, 1998).

**Figure 3:**
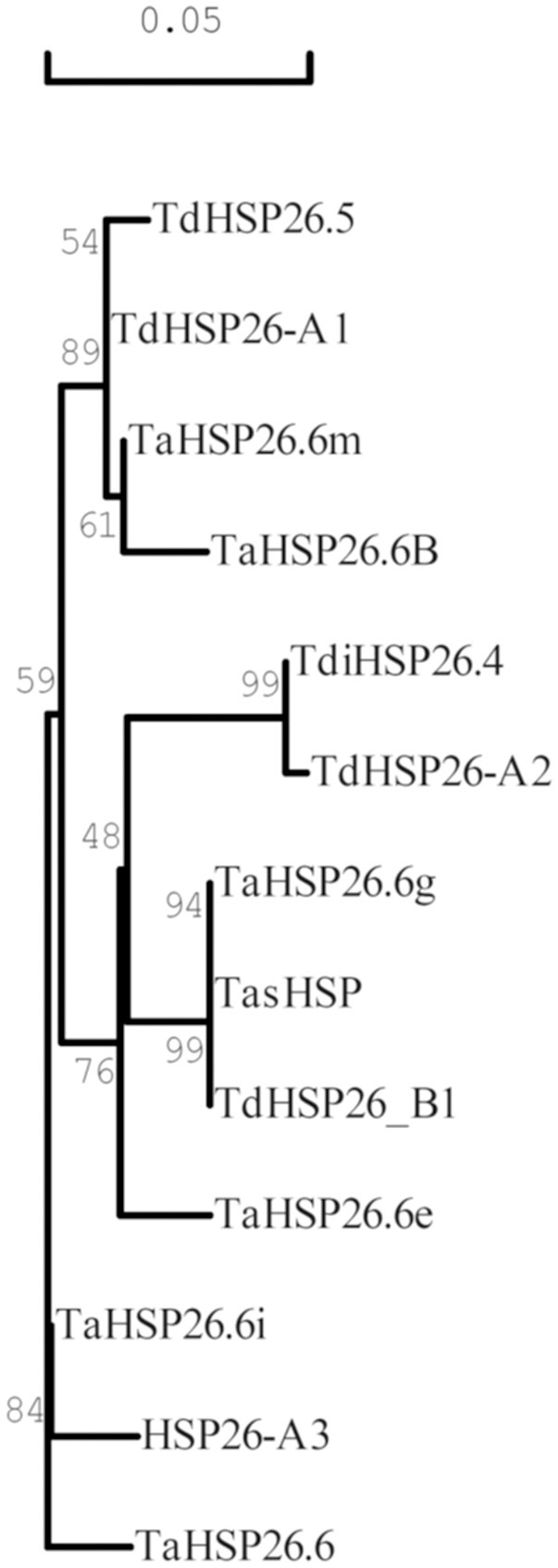
Phylogenetic tree of Triticum HSP26 proteins. The tree was derived by Neighbor Joining methods with bootstrap analysis (1,000 replicates) from the alignment of the entire protein sequence of wheat HSP26 annotated in NCBI database with the newly identified TdHSP-A1, TdHSP26-A2, TdHSP26-A3, and TdHSP26-B1 protein sequences. Accession number are indicated in parenthesis: TaHSP26.6g (AAAC96315), TasHSP (ADN97108), TaHSP26.6i (AAC96316), TdHSP26.5 (CAI96515), TaHSP26.6m (AAC96317), TaHSP26.6B (CAA47745), TaHSP26.6e (AAC96314), TaHSP26.6 (CAA41219), TdiHSP26.4 (CAI96512). Numbers at branch points represent average identities.

The clustering results reported were also confirmed with the phylogenetic tree developed with the Neighbor Joining method using the same multiple sequence alignment (Figure 3).

On the basis of their predicted molecular weights: TdHSP26-A1Ch = 26.5 KDa, -A2Ch = 26.4 KDa, -A3 = 26.6 KDa, -B1Ch = 26.8 KDa (Table 3) and comparing previously isolated *sHsp26* sequence of wheat, the isoforms identified in this paper were considered, respectively, TdHSP26.5, TdHSP26.4, HSP26.6 and TdHSP26.8. A survey of the encoding genes and of other homologous sequences are reported in Table 4.

### *TdHsp26* gene expression analysis

**Figure 4:**
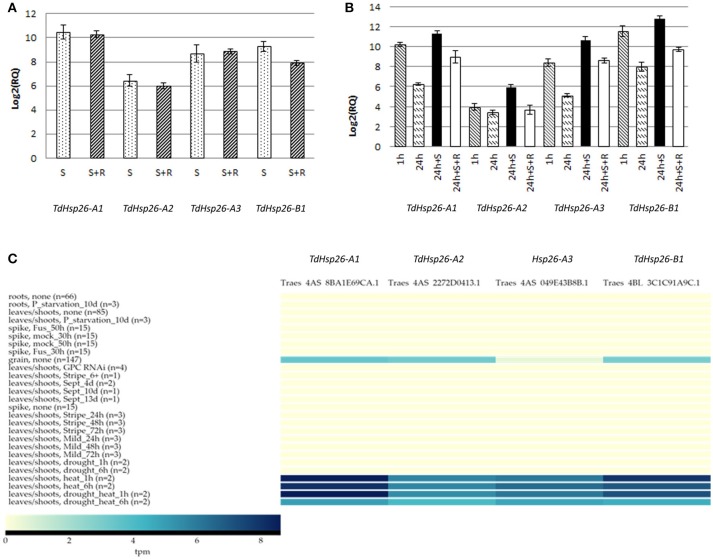
Expression analysis of *TdHsp26-A1Ch, TdHsp26-A2Ch, TdHsp26-A3Ch*, and *TdHsp26-B1Ch* genes in seedling exposed to heat stress. **(A)** Direct heat stress experiment; conditions: 42°C for 2 h (S) and recovery at 25°C for 2 h (S+R). **(B)** Heat acclimation experiment; conditions: 34°C for 1 h (1 h) and 24 h (24 h), stress at 42°C for 2 h (24 h+S), recovery at 25°C for 2 h (24 h+S+R). The induction levels are measured as the fold change (RQ) of the treated samples in respect to the controls and reported as log2(RQ) in the chart **(A,B)**. Bars indicate the standard deviation. **(C)** Heat map of the expression analyses performed with ExpVIP database. The transcript abundances in leaves/shoots after 1 h/6 h heat stress, drought stress, the combination of heat and drought, and the basal expression in grain tissue in no stress condition is reported. Transcript abundances are expressed in log_2_(tpm, transcript per million); (n) indicates the number of RNA-seq samples included in each dataset.

The expression analysis of the four genes was performed on the cDNA obtained from *T. durum* cv. Cham1 seedlings exposed to direct stress (Figure 4A) or to heat acclimation (Figure 4B). All genes were significantly upregulated following a direct heat stress with a range from 1,425-fold for the *TdHsp26-A1* to 87-fold for *TdHsp26-A2* (Figure 4A, Supplementary Table S5A). *TdHsp26-A3* and *TdHsp26-B1* showed an intermediate upregulation: 416- and 620-fold respectively. For the -A genes the upregulation during the recovery phase remained high: 1,245, 64, and 470 fold changes for -*A1, -A2, -A3*, respectively (Figure 4A, Supplementary Table S5A). The expression of the *-B1*, even if still of high levels, dropped to 240-fold during the recovery phase (Figure 4A, Supplementary Table S5A). On the basis of these results, a more detailed analysis of the gene expression following heat acclimation of different durations prior the stress imposition was performed. *TdHsp26-A1* and *-A3* were strongly upregulated 1,205 and 344-fold respectively, after 1 h acclimation reaching levels as in direct stress (Figure 4B, Supplementary Table S5B). *TdHsp26–A2* after 1 h acclimation reached a value lower (15, 8-fold increase) than that of the direct stress (Figure 4B, Supplementary Table S5B). The *-B1* gene has the highest upregulation: 3054-fold (Figure 4B, Supplementary Table S5B). For all genes the expression dropped dramatically after 24 h of acclimation (Figure 4B, Supplementary Table S5B), and the expression level of all genes considerably increased when the heat stress is imposed after acclimation as previously reported for HSP101 (Rampino et al., 2009) with the highest induction for *TaHsp26-B1* (7,197-fold) and *-A1* (2,607-fold) (Figure 4B, Supplementary Table S5B). During recovery the relative expression dropped significantly, but among all the genes analyzed *-B1* remained significantly higher: 852-fold (Figure 4B, Supplementary Table S5B). Differences in the regulation of different members within the same family can be ascribed to some differences in the promoters. To confirm this hypothesis, 1,400 bp of the promoter sequence of all genes have been compared. This analysis reveals some differences in the *cis-acting* elements, particularly in the number of CCAATBOX1 which has been showed to play a key role in the regulation of heat shock genes (data not shown) (Khurana et al., 2013).

The results obtained *in vitro* were confirmed *in silico* blasting the ExpVIP platform (Borrill et al., 2016), but widening the analyses to multiple stresses and tissues. The output reveals that, under non stress conditions, all *TdHsp26* were not expressed and only a low abundance was detected in the grain tissue (Figure 4C). However, the effect of exposing seedlings to high temperature (40°C) stress is to differentially up-regulate the three *TdHsp26* genes: *TdHsp26-A1* and *-B1* are induced in the leaf and shoot to reach a log_2_(tpm) of 8.57 and 7.94, respectively, after a 1 h exposure, and this level is retained for at least a further 5 h (Figure 4C, Supplementary Table S5C). *TdHsp26-A2* is less strongly induced, reaching a log_2_(tpm) of 5.7 after 1 h, with the transcript's abundance remaining steady for at least a further 5 h (Figure 4C, Supplementary Table S5C), as noted previously (Rampino et al., 2009; Abu-Romman, 2016). Drought stress did not induce any of the *TdHsp26* genes, but when combined with high temperatures, *TdHsp26-A1* transcript abundance peaked at a log_2_(tpm) of 8.62 after 1 h, which dropped to 5.0 over the following 5 h (Figure 4C, Supplementary Table S5C); *TdHsp26-B1* behaved very similarly, peaking at 7.19 after 1 h and receding to 4.5 over the following 5 h, while the abundance of *-A2* transcript reached 5.48 after 1 h, but receded to 4.1 over the following 5 h (Figure 4C, Supplementary Table S5C). These results suggest that different family members of *TdHsp26* may have a different relevance in the stress response.

### *De novo TdHsp26* alleles revealed by tilling

**Figure 5:**
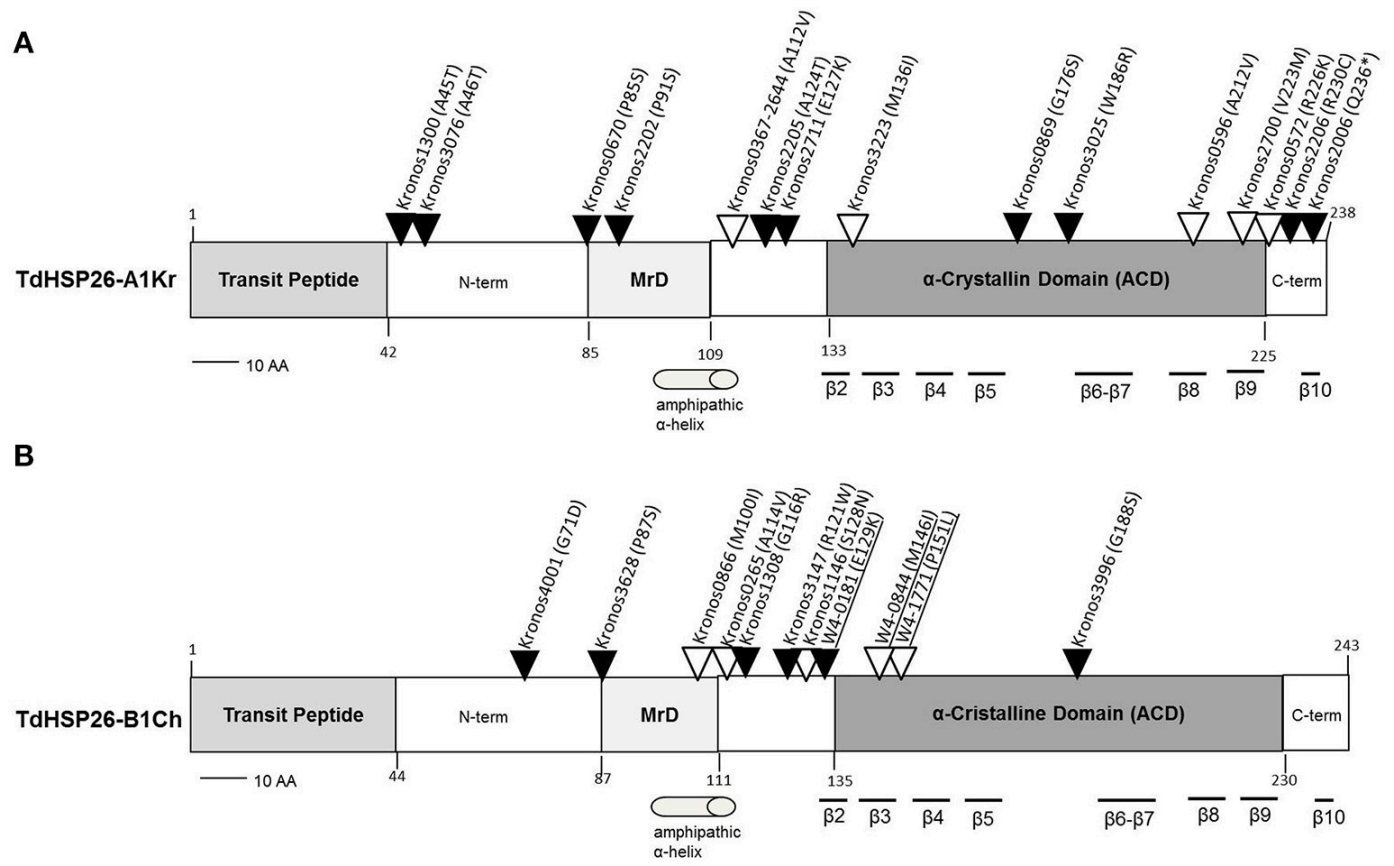
Map of the mis-sense mutations detected in the *TdHsp26-A1Kr* **(A)** and *TdHsp26-B1Ch* **(B)** products. Mutations detected by HRM are underlined. Mutation potentially leading to drastic changes in the protein function are indicated with black arrows and the ones characterized by similar polarity with empty arrows. MrD, Methionine-rich Domain.

On the basis of the expression data *TdHsp26-A1* and *TdHsp26-B1* were selected for the identification of allele variation. Two approaches for allele mining have been used for the identification of SNPs variant on the two homeologous genes *TdHsp26-A1* and *TdHsp26-B1*: firstly, *in silico* high throughput approach and secondly *in vitro* PCR-based HRM screening. The former took advantage of the recently published wheat TILLING database (Krasileva et al., 2017) to find mutations in the Exome sequenced cv. Kronos TILLING population (Parry et al., 2009; King et al., 2015; Krasileva et al., 2017); the latter was performed on cv. Cham1 TILLING population (Uauy et al., 2009). The sequences of the *TdHsp26-A1* and*-B1* alleles present in cv. Cham1 (*-Ch* alleles) were first compared to those present in cv. Kronos (*-Kr* alleles) (Table 4). The two *TdHsp26-A1* alleles (LT220905 and LT220906*)* differ at only two nucleotide positions, one (C217G) in the first exon and the other (A677G) in the second exon; the latter was responsible for a Q73E substitution in the gene product. There were no polymorphisms in *TdHsp26-B1* (LT220911). The wheat TILLING database allowed to identify 28 mutations in *-A1Kr* (Figure 5A, Supplementary Table S2) and 15 in -*B1Ch* (Figure 5B, Supplementary Table S3). Of the 28 *-A1Kr* mutants, one lay within the intron, eleven of the exonic mutations were synonymous and 16 were mis-sense mutations. One truncation (Q236^*^) mutant was detected in the C terminal domain and two mis-sense mutations were identified in the MrD domain (P91S, P85S). Five mis-sense mutations were detected in the ACD: one located in the determinant of the β2 sheet (M136I), one upstream of the β6 (W186R) and one in each of the β8 and β9 sheets (A212V and V223M). One mutation (A112V) was present in two independent lines (Figure 5, Supplementary Table S2). A further two mutations affected the C terminal region (R230C, R226K) and six the N terminal region (Figure 5, Supplementary Table S2). For the –*B1Ch* sequence, three of the 15 mutations were intronic, four were synonymous and eight were mis-sense mutations; the mis-sense mutations were located in the N terminal domain (six: G71D, P87S, A114V, G116R, R121W, S128N), the MrD (one: M100I), and the ACD (one: G188S) (Figure 5B, Supplementary Table S3).

The set of mutations were considered in the light of the PSSM (Position Specific Scoring Matrix) and SIFT (Sorting Intolerant from Tolerant) indices, which predict the mutation's effect on protein function (Kumar et al., 2009). On these basis, a selection cut-off PSSM > 10 and SIFT <0.05 was imposed to select the putative mutants for the *in vivo* confirmation. In addition, the type of SNP, its position in the sequence and the predicted alteration in the protein function (Supplementary Tables S2, S3) were also considered. A comparison between the newly identified sequences with previously annotated sHSP26s of *Triticum* spp. and dicots (Supplementary Figure S3) was done.

For TdHSP26-A1Kr, the P85S, and R230C substitutions identified in the Kronos0670 and Kronos 2206 mutant lines, respectively (Figure 5A, Supplementary Table S2), which involve highly conserved residues, will probably cause a severe alteration on the protein's function. The same could be conceived for the mutant lines Kronos0866 (Figure 4B, Supplementary Table S3) in TdHSP26-B1Ch where an M residue in the position 100, highly conserved between mono- and dicotyledonous species, is substituted by an I.

In the HRM-based search for *de novo* mutants in *TdHsp26-B1Ch*, attention was focused on the MrD and ACD regions. The scan identified eight additional mutations, five of which were in the homozygous and three in the heterozygous state (Supplementary Table S4). All of the mutations were transitions, five were synonymous and three were mis-sense mutations. The latter were M146I and P151L in the ACD, and E129K in the MrD, and each was predicted by PARSESNP to have a substantial effect on the protein's function. Both the M146I (within the β3 sheet) and P151L (CRII region) alleles are of interest, as both the methionine and proline residues are highly conserved across both mono- and dicotyledonous species (Figure 5B, Supplementary Figure S3).

### KASP marker development

Specific KASP markers' primer pairs for each gene copy and polymorphism (King et al., 2015) were developed to successfully target seven of the cv. Kronos *TdHsp26-A1*, three of the cv. Kronos *TdHsp26-B1* and three of the cv. Cham1 *TdHsp26-B1* mutants (Table 5). All the SNPs were subjected to KASP analysis to evaluate the distribution of the FAM and HEX fluorescence signals and to confirm the predicted level of zygosity by comparing the signal distribution of the mutants with the wild type Kronos and Cham1 plants. Every M_3_ individual sampled from the cv. Cham1 lines W4-1771, W4-0181, and W4-0844 and every M_4_ individual sampled from the cv. Kronos lines 0866, 1308, 0265, 2202, 2205, 0869, 0367 carried the mutant allele in the homozygous state. However, based on continued segregation between the wild type and the mutant allele, the Kronos lines 0670, 2206 and 2006 were all predicted to be heterozygous (Table 5). Our results confirm the validity of KASP markers as useful and rapid genotyping tools. The KASP markers developed are currently in use for the selection of the desired mutation in the backcross steps.

**Table 5:** KASP marker analysis of 13 selected *TdHsp26* mutant lines.

**Mutants line**	**Mutation**	**Predicted zygosity**	**No. plants**	**Confirmed zygosity**			**Primer pairs used for the KASP Assay**
				Hom^*^	**Het^&^**	**WT^$^**	
Cham1 W4-0844	G536A	Hom	15	15	0	0	C0844-Fw1/C0844-Fw2/C0844_1771-Rev
Cham1 W4-1771	C550T	Hom	11	11	0	0	C1771-Fw1/C1771-Fw2/C0844_1771-Rev
Cham1 W4-0181	G483A	Hom	13	13	0	0	C0181-Fw/C0181-Rev1/C0181-Rev2
Kronos0866	G398A	Hom	15	15	0	0	K0866-Fw1/K0866-Fw2/K0866-Rev
Kronos1308	G444A	Hom	13	13	0	0	K1308-Fw1/K1308-Fw2/K1308_0265-Rev
Kronos0265	C439T	Hom	15	15	0	0	K0265-Fw1/K0265-Fw2/K1308_0265-Rev
Kronos2202	C361T	Hom	16	13	0	0	K2202-Fw/K2202-Rev1/K2202-Rev2
Kronos0670	C253T	Het	16	3	8	5	K0670-Fw1/K0670-Fw2/K0670-Rev
Kronos2205	G460A	Hom	15	15	0	0	K2205-Fw/K2205-Rev1/K2205-Rev2
Kronos2206	C778T	Het	15	6	3	6	K2206-Fw/K2206-Rev1/K2206-Rev2
Kronos2006	C796T	Het	11	3	3	5	K2006-Fw1/K2006-Fw2/K2006-Rev
Kronos0869	G616A	Hom	16	15	0	0	K0869-Fw1/K0869-Fw2/K0869-Rev
Kronos0367	C425T	Hom	15	15	0	0	K0367-Fw1/K0367-Fw2/K0367-Rev

*Between 11 and 16 individuals in the M_3_ (cv. Cham1) or M_4_ (cv. Kronos) generation were sampled. The zygosity predicted with the TILLING online resource and the zygosity verified in vivo in each plant of the mutant lines is indicated*.

* Homozygous;

& Heterozygous;

$ *Wild Type*.

## Discussion

The recognition of the biological significance of the class of proteins long referred to as “heat shock proteins” (Key et al., 1981; Altschuler and Mascarenhas, 1982; Nguyen and Joshi, 1994) has prompted an extensive effort to correlate individual HSPs with plant tolerance to high temperature stress. Here, sequence variation in the *T. durum* family of HSP26 proteins was explored. The choice of target was based on the known protective role played by sHSPs such as HSP26 over photosynthesis and the synthesis and compartmentalization of key metabolites in plants challenged by high temperature stress (Maestri et al., 2002; Chauhan et al., 2012; Khurana et al., 2013). The s*Hsp26* family was represented by four functional genes, three mapping to a single A genome chromosome and one to its B genome homoeologue. The predicted products displayed a typical sHSP topology, sharing a very high level of sequence similarity with other wheat sHSPs. Of the four gene products, three have been previously detected in cultivated wheat, but the only known match to TdHSP26-A2 is a protein present in the progenitor *T. dicoccoides*.

Despite the presence of a long intron within the –*A3* sequence that hampered the isolation of the full genomic sequence, the gene was correctly transcribed. Moreover, the sequence alignment with the other *TdHsp26* members does not allow to conclude the presence of pseudogenes within the *TdHsp26* family.

*TdHsp26* genes were differently regulated upon direct heat stress and especially after acclimation. In particular, *TdHsp26-A1* showed the highest upregulation following direct heat stress and *TdHsp26-B1* the highest one when the heat stress was imposed after acclimation. *TdHsp26-A2* showed the lowest expression level but was clearly induced following 1 h acclimation and with a 46% increase after 24 h acclimation and stress. All these data confirm an active role of *sHsp26* in acquired thermotolerance and suggest that a different role may be played by different members of the same family (Comastri, 2016). This supports the hypothesis that allelic variability exists within the same gene, which can greatly support our current work on different newly identified and isolated mutants. The *in vitro* transcriptomic analysis was extended to other tissues and to stress conditions by querying the *in silico* ExpVIP database. For instance, *TdHsp26* genes were strongly up-regulated by high temperature but even more by a combination of high temperature and drought, suggesting a role of sHSPs in both stress responses (Al-Whaibi, 2011; Rampino et al., 2012; Khurana et al., 2013; Liu et al., 2015).

Various authors proposed that plant thermotolerance could be enhanced via transgenesis by either over-expressing native *Hsp* genes or introducing heterologous homologs (Bita and Gerats, 2013). Recent developments in genome editing (Bortesi and Fischer, 2015; Osakabe and Osakabe, 2015) offer the opportunity to specifically alter the sequence of native gene copies with the intention of improving the effectiveness of their products.

Transgenic approaches have been extensively tested in the model species *Arabidopsis* and *Nicotiana* spp., but to a lesser degree in crop species (Cheng, 2009; Wang et al., 2011; Calestani et al., 2015). Gene editing is in principle applicable to any organism, but as yet has not been attempted with a view to improve crop thermotolerance, since the genetic basis of the trait is still unclear. Gene editing platforms are intended to alter a specific sequence, leaving the background genome unaffected. Some off-target effects are known, however, to compromise CRISPR/Cas9 specificity (Bortesi and Fischer, 2015; Song et al., 2016), while the RNAi approach can also randomly disrupt non-target genes (Jones, 2015). A major attraction of the mutagenesis/TILLING (Henikoff et al., 2004; Wang et al., 2008, 2010, 2014; Prohens, 2011) approach is that it does not rely on transgenesis and has received high public acceptance and is exempted from the biosafety regulations imposed on transgenics (Tadele, 2016). In addition, a wide range of truncations but also missense mutations are detectable. Its application in wheat is complicated by the need to deal with multiple gene copies even for single gene targets (Borrill et al., 2015).

The results presented here, highlight that TILLING is applicable also to durum wheat, a plant with a high genome complexity (Krasileva et al., 2017). The recovery of a number of mutations open new perspectives in molecular breeding for wheat tolerance. Different mutations with positive effects on the protein synthesis can be accumulated within the same genotype with an *in vivo* pyramidization.

Indeed, for a multigene family as the *sHsp26* composed by 4 genes, what has to be expected is an additive effect which can significantly vary the heat stress response.

TILLING, as with any mutagenesis programme, requires the background cleaning of unwanted mutations via backcrossing. Previously, papers successfully reported the use of TILLING in wheat for the identification of mutants for starch related genes, pathogen related genes and vernalization response (for review see Kumar et al., 2010) where mutant detection was achieved by HRM (Ishikawa et al., 2010; Botticella et al., 2011). So far, to best of our knowledge, the applicability of TILLING for abiotic stress genes in allele mining for multigene families remains an open issue.

In this paper we have developed genome specific primer(s) which successfully target a multigene family, this opened the possibility to extent the TILLING approach also to this complex gene family. In this paper the characterization of four functional members of the complex multigene family of *sHsp26* in wheat and the identification of mutants potentially involved in heat adaptation have been achieved. A total of 27 mis-sense mutations have been identified by coupling TILLING and HRM, of which 11 affected either the MrD or the ACD, both of which are important for substrate binding and oligomerization. We believe this is the first set of small HSP mutants available in durum wheat.

Next step of the research will be the phenotypic characterization of the mutant lines in order to link the specific mutation to the heat stress adaptation. At this purpose, a backcross program is ongoing to obtain NILs (Near Isogenic Lines) to be used for thermotolerance tests and for the phenotyping in controlled environment. The possibility to access to a wide range of variability within a complex gene family, opens new perspectives for genotype to phenotype association.

Due to the polyploidy genome, gene duplication in wheat often limits the use of a forward genetics as the effect of single-gene knockouts is frequently masked at functional level due to a redundancy of the homoeologous genes present in the other genomes. To overcome this drawback, the combination of multiple homoeologous mutations in the same background was obtained by crossing single genotype carrying single mutations on A and B genome respectively to pyramid the mutations.

The development of specific KASP markers for the mutation is therefore mandatory to follow the mutation within generations, either in the case of single backcrosses or in natural pyramiding of the genes.

Taking all together these results provide a wider view on the contribution of sHsps in the stress response and open new perspectives for the application of the TILLING strategy, not only focused to the coding sequence but also to the promoter region for functional genetics studies, to better understand the mechanisms that take place during the thermotolerance development in wheat.

## Author contributions

AC carried out the gene isolation and characterization, the TILLING analysis, and the characterization of mutants. The manuscript was drafted by AC, MJ, and NM. CU and JS provided the mutagenized wheat populations. AC and JS developed and tested the KASP markers. DP, CU, HN, and NM edited the manuscript. NM and MJ coordinated the work in Parma, CU coordinated the work in Norwich.

### Conflict of interest statement

The authors declare that the research was conducted in the absence of any commercial or financial relationships that could be construed as a potential conflict of interest.
