# Supplementary material for: Heat in Wheat: Exploit Reverse Genetic Techniques to Discover New Alleles Within the Triticum durum sHsp26 Family
Source: Front Plant Sci. 2018 Sep 19;9:1337. doi: 10.3389/fpls.2018.01337 (PMC6156267; doi:10.3389/fpls.2018.01337)
Supplement: Supplementary file 1 [file Table_1.PDF]

Supplementary Material

Heat in wheat: exploit reverse genetic techniques to discover new alleles within the *Triticum durum* sHsp26 family

Alessia Comastri, Michela Janni[*], James Simmonds [4], Cristobal Uauy[4], Domenico Pignone[2], Henry T. Nguyen[5], Nelson Marmiroli[1].

*** Correspondence:** Corresponding Author: michela.janni@ibbr.cnr.it

Supplementary Table S1 Primer sequences used for the KASP assays. Three primers were used for each assay: one was a common primer and the other two were allele-specific, carrying either a FAM (5'-GAAGGTGACCAAGTTCATGCT) or a HEX (5'-GAAGGTCGGAGTCAACGGATT) tail at their 5' end.

Primer name	Type	Sequence 5'-3'
C0844-Fw1	Forward_WT	GGACGACAAGGAGGTGAAGAT**G-FAM**
C0844-Fw2	Forward_Mut	GGACGACAAGGAGGTGAAGAT**A-VIC/HEX**
C0844_1771-Rev	Reverse_Common	ACCATCACCTTCACCTCCTC
C1771-Fw1	Forward_WT	AGATGCGGTTCGACATGC**C-FAM**
C1771-Fw2	Forward_Mut	AGATGCGGTTCGACATGC**T-VIC/HEX**
C0181-Fw	Forward_Common	GGACGATGCGGCAGATGCTT
C0181-Rev1	Reverse_Mut	ACGGCATCCGCGGCATCT**T-FAM**
C0181-Rev2	Reverse_WT	ACGGCATCCGCGGCATCT**C-VIC/HEX**
K0866-Fw1	Forward_WT	TGTCGCCGATGAGGACGAT**G-FAM**
K0866-Fw2	Forward_Mut	TGTCGCCGATGAGGACGAT**A-VIC/HEX**
K0866-Rev	Reverse_Common	CAGCCGGTCCATCGTGTCA
K1308-Fw1	Forward_WT	CTGTTCGACGACGCCGTG**G-FAM**
K1308-Fw2	Forward_Mut	CTGTTCGACGACGCCGTG**A-VIC/HEX**
K1308_0265-Rev	Reverse_Common	ATCTCGCTCGCCGCCGCCA
K0265-Fw1	Forward_WT	ACCGGCTGTTCGACGACG**C-FAM**
K0265-Fw2	Forward_Mut	ACCGGCTGTTCGACGACG**T-VIC/HEX**
K2202-Fw	Forward_Common	GAGCATGACGGTTTACTTCAAC
K2202-Rev1	Reverse_WT	CCTCATCGGCGACATCG**G-FAM**
K2202-Rev2	Reverse_Mut	CCTCATCGGCGACATCG**A-VIC/HEX**
K0670-Fw1	Forward_WT	CGCTGGATTTGACATCTCC**C-FAM**
K0670-Fw2	Forward_Mut	CGCTGGATTTGACATCTCC**T-VIC/HEX**
K0670-Rev	Reverse_Common	CAAAATTCCGTGTAAAAGAACATCA
K2205-Fw	Forward_Common	GACGATGCGGCAGATGCTG
K2205-Rev1	Reverse_WT	GGCGTCTCGCTCGCCG**C-FAM**
K2205-Rev2	Reverse_Mut	GGCGTCTCGCTCGCCG**T-VIC/HEX**
K2206-Fw	Forward_Common	AGCTCCTACGGCATGCGA
K2206-Rev1	Reverse_WT	CACGTCGATGACCTTGC**G-FAM**
K2206-Rev2	Reverse_Mut	CACGTCGATGACCTTGC**A-VIC/HEX**
K2006-Fw1	Forward_WT	GCAAGGTCATCGACGTG**C-FAM**
K2006-Fw2	Forward_Mut	GCAAGGTCATCGACGTG**T-VIC/HEX**
K2006-Rev	Reverse_Common	GGGTACAGAGTCTCGCACAA
K0869-Fw1	Forward_WT	AGCACAAGAAGGAGGCC**G-FAM**
K0869-Fw2	Forward_Mut	AGCACAAGAAGGAGGCC**A-VIC/HEX**
K0869-Rev	Reverse_Common	CTCTTGTCGCACTCGTCC
K0367-Fw1	Forward_WT	CCGGCTGTTCGACGACG**C-FAM**
K0367-Fw2	Forward_Mut	CCGGCTGTTCGACGACG**T-VIC/HEX**
K0367-Rev	Reverse_Common	CCTCCTTCTCGTCCTCCATA
