# Supplementary material for: Heat in Wheat: Exploit Reverse Genetic Techniques to Discover New Alleles Within the Triticum durum sHsp26 Family
Source: Front Plant Sci. 2018 Sep 19;9:1337. doi: 10.3389/fpls.2018.01337 (PMC6156267; doi:10.3389/fpls.2018.01337)
Supplement: Supplementary file 2 [file Table_2.PDF]

Supplementary Material

Heat in wheat: exploit reverse genetic techniques to discover new alleles within the *Triticum durum* sHsp26 family

Alessia Comastri, Michela Janni[*], James Simmonds [4], Cristobal Uauy[4], Domenico Pignone[2], Henry T. Nguyen[5], Nelson Marmiroli[1].

*** Correspondence:** Corresponding Author: michela.janni@ibbr.cnr.it

Supplementary Table S2 Cv. Kronos derivative lines carrying a mutation on *TdHsp26-A1* retrieved on the wheat TILLING database using TRAES_4AS_8BA1E69CA as query.

Line	Chromosome position	Position on *TdHsp26-A1Kr* gene[a]	Confidence[d]	Het/Hom	Consequence	Nucleotide change	Position on gene structure[a]	Amino Acid change[a]	Domain[b]	PSSM[c]	SIFT[c]
Kronos1300	48083357	133	high	het	missense_variant	Gcg/Acg	Ex I	A45T	N-term		0.04
Kronos3076	48083354	136	medium	het	missense_variant	Gcg/Acg	Ex I	A46T	N-term		1.0
Kronos2193	48083349	141	high	het	synonymous_variant	caG/caA	Ex I	Q47=	N-term		
Kronos1179	48083340	150	low	het	synonymous_variant	agA/agG	Ex I	R50=	N-term		
Kronos3004	48083307	183	high	het	synonymous_variant	gcC/gcT	Ex I	A61=	N-term		
Kronos1179	48083298	192	low	het	synonymous_variant	gcT/gcC	Ex I	A64=	N-term		
Kronos2576	48083283	207	high	het	synonymous_variant	ggC/ggT	Ex I	G69=	N-term		
Kronos0670	48083237	253	high	het	missense_variant	Ccg/Tcg	Ex I	P85S	MrD	16.4	0.09
Kronos2488	48083220	270	high	het	intron variant		INTRON				
Kronos2202	48083129	361	high	hom	missense_variant	Ccg/Tcg	Ex II	P91S	MrD	19.7	0.01
Kronos2644	48083065	425	high	het	missense_variant	gCc/gTc	Ex II	A112V	N-term		0.05
Kronos0367	48083065	425	high	hom	missense_variant	gCc/gTc	Ex II	A112V	N-term		0.05
Kronos0877	48083064	426	high	het	synonymous_variant	gcC/gcT	Ex II	A112=	N-term		
Kronos3913	48083055	435	high	het	synonymous_variant	ttC/ttT	Ex II	F115=	N-term		
Kronos2205	48083030	460	high	hom	missense_variant	Gcg/Acg	Ex II	A124T	N-term		0.29
Kronos2711	48083021	469	high	het	missense_variant	Gag/Aag	Ex II	E127K	N-term	0	0.92
Kronos3223	48082992	498	high	het	missense_variant	atG/atA	Ex II	M136I	ACD	13.4	0.15
Kronos3742	48082974	516	high	hom	synonymous_variant	Gtg/gtA	Ex II	V142=	ACD		
Kronos3052	48082899	591	high	het	synonymous_variant	atC/atT	Ex II	I167=	ACD		
Kronos0869	48082874	616	high	hom	missense_variant	Ggc/Agc	Ex II	G176S	ACD		0.53
Kronos3025	48082844	646	low	het	missense_variant	Tgg/Agg	Ex II	W186R	ACD		0.39
Kronos0243	48082830	660	high	het	synonymous_variant	cgC/cgT	Ex II	R190=	ACD		
Kronos0852	48082773	717	high	hom	synonymous_variant	caG/caA	Ex II	Q209=	ACD		
Kronos0596	48082765	725	high	het	missense_variant	gCc/gTc	Ex II	A212V	ACD	8.7	0.0
Kronos2700	48082733	757	high	hom	missense_variant	Gtg/Atg	Ex II	V223M	ACD	6.3	0.01
Kronos0572	48082723	767	high	het	missense_variant	aGg/aAg	Ex II	R226K	C-term	1.4	0.78
Kronos2206	48082712	778	high	het	missense_variant	Cgc/Tgc	Ex II	R230C	C-term	12.5	0.00
Kronos2006	48082694	796	high	het	stop_gained	Cag/Tag	Ex II	Q236*	C-term		

[a] Position of the mutation on the *TdHsp26-A1Kr* (LT220906) gene and with respect to gene structure, the residue changes were verified manually for each mutation.

[b] The location of the mutation with respect to the domain was predicted on the basis of sequence alignment and literature information regarding HSP26 structure.

[c] PSSM and SIFT values calculated using PARSESNP software; those predicted to have a significant effect on protein function are shown in red.

[d] The confidence score of the expected mutation on the basis of the exome capture sequencing data predicted with online TILLING resource (coverage of 4 is low, 5 is medium, 6 is high)
