# Supplementary material for: Heat in Wheat: Exploit Reverse Genetic Techniques to Discover New Alleles Within the Triticum durum sHsp26 Family
Source: Front Plant Sci. 2018 Sep 19;9:1337. doi: 10.3389/fpls.2018.01337 (PMC6156267; doi:10.3389/fpls.2018.01337)
Supplement: Supplementary file 3 [file Table_3.PDF]

Supplementary Material

Heat in wheat: exploit reverse genetic techniques to discover new alleles within the *Triticum durum* sHsp26 family

Alessia Comastri, Michela Janni*, James Simmonds [4], Cristobal Uauy[4], Domenico Pignone[2], Henry T. Nguyen[5], Nelson Marmiroli[1].

*** Correspondence:** Corresponding Author: michela.janni@ibbr.cnr.it

Supplementary Table S3 Cv. Kronos derivative lines carrying a mutation on TdHsp26-B1 retrieved on the wheat TILLING database using Traes_4BL_3C1C91A9C as the query sequence.

Line	Position on *TdHsp26-B1Ch* gene[a]	Confidence[d]	Het/Hom	Consequence	Nucleotide change	Position on gene structure[a]	Amino Acid change[a]	Domain[b]	PSSM[c]	SIFT[c]
Kronos4001	212	low	het	missense_variant	gGc/gAc	Ex I	G71D	N-term	15.3	0.01
Kronos3628	259	high	het	missense_variant	CcG/Tcg	Ex I	P87S	N-term	15.5	0.13
Kronos2574	266	high	hom	intron variant		INTRON				
Kronos3113	301	high	het	intron variant		INTRON				
Kronos0323	336	high	het	intron variant		INTRON				
Kronos0866	398	medium	hom	missense_variant	atG/atA	Ex II	M100I	MrD	15.5	0.03
Kronos0265	439	medium	hom	missense_variant	gCc/gTc	Ex II	A114V	N-term	9.1	0.28
Kronos1308	444	high	hom	missense_variant	Ggg/Agg	Ex II	G116R	N-term		0.11
Kronos3487	446	high	het	synonymous_variant	ggG/ggA	Ex II	G116=	N-term		
Kronos3147	459	high	het	missense_variant	Cgg/Tgg	Ex II	R121W	N-term		0.02
Kronos3186	464	medium	het	synonymous_variant	cgC/cgT	Ex II	R122=	N-term		
Kronos1146	481	high	het	missense_variant	aGc/aAc	Ex II	S128N	N-term		0.45
Kronos2293	491	high	het	synonymous_variant	ccG/ccA	Ex II	P131=	N-term		
Kronos3996	660	high	het	missense_variant	Ggc/Agc	Ex II	G188S	ACD		0.24
Kronos1159	713	high	het	synonymous_variant	tgC/tgT	Ex II	C210=	ACD		

[a] Position of the mutation on the *TdHsp26-B1Ch* (LT220911) gene and with respect to gene structure, the residue changes were verified manually for each mutation.

[b] The location of the mutation with respect to domain was predicted on the basis of sequence alignment and literature information regarding HSP26 structure.

[c] PSSM and SIFT values calculated using PARSESNP software; those predicted to have a significant effect on protein function are shown in red.

[d] The confidence score of the expected mutation on the basis of the exome capture sequencing data predicted with online TILLING resource (coverage of 4 is low, 5 is medium, 6 is high)
