# Supplementary material for: Heat in Wheat: Exploit Reverse Genetic Techniques to Discover New Alleles Within the Triticum durum sHsp26 Family
Source: Front Plant Sci. 2018 Sep 19;9:1337. doi: 10.3389/fpls.2018.01337 (PMC6156267; doi:10.3389/fpls.2018.01337)
Supplement: Supplementary file 4 [file Table_4.PDF]

Supplementary Material

Heat in wheat: exploit reverse genetic techniques to discover new alleles within the *Triticum durum* sHsp26 family

Alessia Comastri, Michela Janni[*], James Simmonds [4], Cristobal Uauy[4], Domenico Pignone[2], Henry T. Nguyen[5], Nelson Marmiroli[1].

*** Correspondence:** Corresponding Author: michela.janni@ibbr.cnr.it

Supplementary Table S4 Mutations retrieved in *TdHsp26-B1* identified by HRM in the cv. Cham1

Mutant line	Position on *TdHsp26-B1Ch*	WT_base	Mut_base	Het/Ho	Consequence	Amino Acid	Domain	PSSM	SIFT
W4-0841	425	G	A	Het	synonymous_variant	R109=	N-term		
W4-1842	449	C	T	Hom	synonymous_variant	F117=	N-term		
W4-0181	483	G	A	Hom	missense_variant	E129K	N-term		0.27
W4-1152	500	G	A	Het	synonymous_variant	P134=	N-term		
W4-0844	536	G	A	Hom	missense_variant	M146I	ACD	18.4	0.02
W4-1771	550	C	T	Hom	missense_variant	P151L	ACD	25.6	0.00
W4-0940	554	G	A	Hom	synonymous_variant	G152=	ACD		
W4-2013	563	G	A	Het	synonimous variant	R155=	ACD		
