# Supplementary material for: Heat in Wheat: Exploit Reverse Genetic Techniques to Discover New Alleles Within the Triticum durum sHsp26 Family
Source: Front Plant Sci. 2018 Sep 19;9:1337. doi: 10.3389/fpls.2018.01337 (PMC6156267; doi:10.3389/fpls.2018.01337)
Supplement: Supplementary file 5 [file Table_5.PDF]

Supplementary Material

Heat in wheat: exploit reverse genetic techniques to discover new alleles within the *Triticum durum* sHsp26 family

Alessia Comastri, Michela Janni[*], James Simmonds [4], Cristobal Uauy[4], Domenico Pignone[2], Henry T. Nguyen[5], Nelson Marmiroli[1].

*** Correspondence:** Corresponding Author: michela.janni@ibbr.cnr.it

Supplementary Table S5. Expression analysis of TaHsp26 genes following (A) direct heat stress; (B) heat acclimation experiments (C) combined heat and drought stress. (A) Direct heat stress performed on 10-days seedling of *T. durum* Cv. Cham1: Ctrl, unstressed control grown at light/dark 16h/8h with 25°C/20°C for 10 days; S, direct stress for 2 hours at 42°C; S+R, S followed by recovery at 25°C for 2 hours. (B) Heat acclimation experiment on 10-days seedling of *T. durum* Cv. Cham1. Ctrl, unstressed control grown at light/dark 16h/8h with 25°C/20°C for 10 days; 1h, acclimation for 1 hour at 34°C; 24h, acclimation for 24 hours at 34°C; 24h+S, acclimation 24h followed by 2 hours stress at 42°C; 24h+S+R, 24h+S followed by recovery at 25°C for 2 hours. (C) Expression analyses performed with ExpVIP database on the sHsp26 transcripts (Traes_4AS_8BA1E69CA.1 for TdHsp26-A1, Traes_4AS_2272D0413 for TdHsp26-A2, Traes_4AS_049E43B8B for TdHsp26-A3, Traes_4BL_3C1C91A9C for TdHsp26-B1). Transcript abundance in grain tissue in no stress condition (grain, none); transcript abundances in leaves/shoots after 1h heat stress (leaves/shoots, 1 hour of heat stress); transcript abundances in leaves/shoots after 6h heat stress (leaves/shoots, 6 hours of heat stress); transcript abundances in leaves/shoots after the combination of heat and drought for 1 (leaves/shoots, 1 hour of drought&heat combined stress); transcript abundances in leaves/shoots after the combination of heat and drought for 6 hours (leaves/shoots, 6 hours of drought&heat combined stress).

(A) Direct heat stress experiment

Gene	Treatment	Ct^	ΔΔCt	RQ*	Log2(RQ)
Actin	Ctrl	22,89			
	S	22,24			
	S+R	22,84			
TdHsp26-A1Ch	Ctrl	36,68	0,00	1,00	
	S	25,55	-10,48	1425,74	10,48
	S+R	26,34	-10,28	1245,49	10,28
TdHsp26-A2Ch	Ctrl	30,99	0,00	1,00	
	S	23,89	-6,45	87,43	6,45
	S+R	24,94	-6,00	64,00	6,00
TdHsp26-A3Ch	Ctrl	33,54	0,00	1,00	
	S	24,19	-8,70	416,59	8,70
	S+R	24,61	-8,88	470,32	8,88
TdHsp26-B1Ch	Ctrl	28,27	0,00	1,00	
	S	18,34	-9,28	620,59	9,28
	S+R	20,30	-7,91	240,52	7,91

Supplementary Table S5. Continue

(B) Heat acclimation experiment

Gene	Treatment	Ct^	ΔΔCt	RQ*	Log2(RQ)
Actin	Ctrl	24,49			
	1h	24,01			
	24h	24,20			
	24h+S	23,44			
	24h+S+R	19,58			
TdHsp26-A1Ch	Ctrl	38,49	0,00	1,00	
	1h	27,78	-10,24	1205,15	10,24
	24h	31,95	-6,26	76,37	6,26
	24h+S	26,09	-11,35	2607,29	11,35
	24h+S+R	24,63	-8,96	496,28	8,96
TdHsp26-A2Ch	Ctrl	30,92	0,00	1,00	
	1h	26,47	-3,98	15,80	3,98
	24h	27,22	-3,41	10,65	3,41
	24h+S	23,94	-5,93	60,90	5,93
	24h+S+R	22,36	-3,65	12,55	3,65
TdHsp26-A3Ch	Ctrl	37,75	0,00	1,00	
	1h	28,85	-8,43	344,49	8,43
	24h	32,37	-5,09	34,02	5,09
	24h+S	26,08	-10,62	1570,13	10,62
	24h+S+R	24,21	-8,63	394,81	8,63
TdHsp26-B1Ch	Ctrl	32,69	0,00	1,00	
	1h	20,64	-11,58	3054,39	11,58
	24h	24,36	-8,03	261,68	8,03
	24h+S	18,82	-12,81	7197,77	12,81
	24h+S+R	18,04	-9,74	852,17	9,74

Supplementary Table S5. Continue

(C) ExpVIP expression data

Gene	Treatment [&]	Log2(tpm[§])	Sem[$]
Traes_4AS_8BA1E69CA.1 (Hsp26-A1)	grain, none(n=147)	3,44	4,46
	leaves/shoots, 1 hour of heat stress(n=2)	8,57	7,57
	leaves/shoots, 6 hour of heat stress(n=2)	8,08	5,56
	leaves/shoots, 1 hour of drought&heat combined stress(n=2)	8,62	5,35
	leaves/shoots, 6 hour of drought&heat combined stress(n=2)	4,97	0
Traes_4AS_2272D0413.1 (Hsp26-A2)	grain, none(n=147)	3,21	4,88
	leaves/shoots, 1 hour of heat stress(n=2)	5,69	4,63
	leaves/shoots, 6 hour of heat stress(n=2)	5,67	0
	leaves/shoots, 1 hour of drought&heat combined stress(n=2)	5,48	1,26
	leaves/shoots, 6 hour of drought&heat combined stress(n=2)	4,12	0
Traes_4AS_049E43B8B.1 (Hsp26-A3)	grain, none(n=147)	0,55	1,34
	leaves/shoots, 1 hour of heat stress(n=2)	6,02	4,88
	leaves/shoots, 6 hour of heat stress(n=2)	6,44	1,6
	leaves/shoots, 1 hour of drought&heat combined stress(n=2)	6,09	4,72
	leaves/shoots, 6 hour of drought&heat combined stress(n=2)	4,78	1,8
Traes_4BL_3C1C91A9C.1 (Hsp26-B1)	grain, none(n=147)	3,04	4,17
	leaves/shoots, 1 hour of heat stress(n=2)	7,94	6,86
	leaves/shoots, 6 hour of heat stress(n=2)	6,99	3,41
	leaves/shoots, 1 hour of drought&heat combined stress(n=2)	7,19	2,59
	leaves/shoots, 6 hour of drought&heat combined stress(n=2)	4,53	1,07

^Ct, Threshold cycle; *RQ Relative quantitation; § tpm, transcript per million; and (n) number of RNA-seq samples included in each dataset; $ Sem (Standard Error)
