# Supplementary material for: Heat in Wheat: Exploit Reverse Genetic Techniques to Discover New Alleles Within the Triticum durum sHsp26 Family
Source: Front Plant Sci. 2018 Sep 19;9:1337. doi: 10.3389/fpls.2018.01337 (PMC6156267; doi:10.3389/fpls.2018.01337)

Supplementary Material

Heat in wheat: exploit reverse genetic techniques to discover new alleles within the *Triticum durum* sHsp26 family

Alessia Comastri, Michela Janni[*], James Simmonds [4], Cristobal Uauy[4], Domenico Pignone[2], Henry T. Nguyen[5], Nelson Marmiroli[1].

*** Correspondence:** Corresponding Author: michela.janni@ibbr.cnr.it

Supplementary Figure S1. Chromosome arm location of the TdHsp26 genes. Lane 1: Nullisomic 4A tetrasomic 4D (N4AT4D), 2: N4DT4B, 3: Ditelosomic 4AS (DT4AS), 4: DT4AL, 5: DT4BS, 6: DT4DS, 7: DT4DL, 8: cv. Cham1, 9: Zero template control. The primer pairs were: A1-9F/A1-4R (*TdHsp26-A1Ch*), A2-24F/A2-18R (*TdHsp26-A2Ch*), A3-20F/A3-29R (*TdHsp26-A3Ch*), B1-PT10F/R (*TdHsp26-B1Ch*), TaACT-Fw/Rev (Actin).

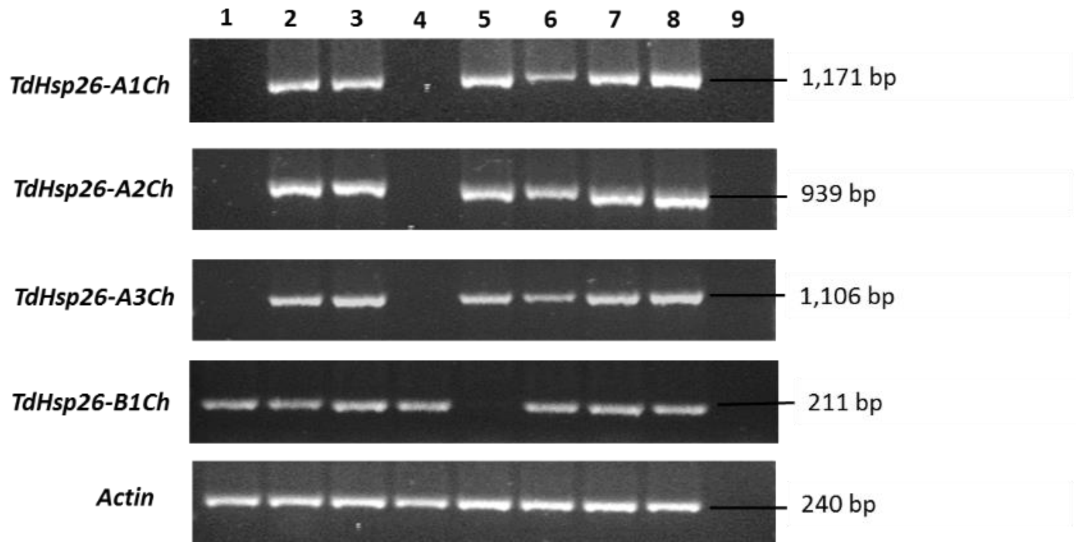

Supplement: Supplementary file 6 [file Image_1.PDF]
