# Supplementary material for: Heat in Wheat: Exploit Reverse Genetic Techniques to Discover New Alleles Within the Triticum durum sHsp26 Family
Source: Front Plant Sci. 2018 Sep 19;9:1337. doi: 10.3389/fpls.2018.01337 (PMC6156267; doi:10.3389/fpls.2018.01337)
Supplement: Supplementary file 7 [file Image_2.PDF]

Supplementary Material

Heat in wheat: exploit reverse genetic techniques to discover new alleles within the *Triticum durum* sHsp26 family

Alessia Comastri, Michela Janni[*], James Simmonds [4], Cristobal Uauy[4], Domenico Pignone[2], Henry T. Nguyen[5], Nelson Marmiroli[1].

*** Correspondence:** Corresponding Author: michela.janni@ibbr.cnr.it

Supplementary Figure S2 Multiple alignment of the *TdHsp26-A1Ch* (LT220905), *-A2Ch* (LT220907), *-A3Ch* (LT220909), *-B1Ch* (LT220911) and *Hsp26-A3* sequences. The putative intron sequences are highlighted in gray. The putative splicing sites predicted with FSPLICE are in bold. The portion of intron II in the *Hsp26-A3* not resolved in the TGAC scaffold, of approximately 3828 bp, is indicated in red on the sequence.

```
TdHsp26-A3Ch    ATGGCTGCAGCGAACGCTCCCTTCGCTCTCGTCAGCCGCC     40
  Hsp26-A3      ATGGCTGCAGCGAACGCTCCCTTCGCTCTCGTCAGCCGCC     40
TdHsp26-A2Ch    ATGGCTGCcGCGAACGCcCCCTTCGCTaTaGTCAGCCGCC     40
TdHsp26-A1Ch    ATGGCcGCAGCGAAtGCcCCCTTCGCTC...TCAGCCGCC     37
TdHsp26-B1Ch    ATGGCTGacGCGAACGCcCCCTTCGCTCTCGTCAGCCGCC     40

TdHsp26-A3Ch    TCTCCCCGGCCGCGCGCCTGCCCATCCGTGCCTGGAGAGC     80
  Hsp26-A3      TCTCCCCGGCCGCGCGCCTGCCCATCCGTGCCTGGAGAGC     80
TdHsp26-A2Ch    TtTCCCCGGCCGCGCGCCTGCCgATCCGTGCCTGGAGgGC     80
TdHsp26-A1Ch    TCTCCCCGGCCGCGCGCCTGCCgtTCCGTGCCTGGAGAGC     77
TdHsp26-B1Ch    TCTCCCCGGCCGCGCGCCTGCCCATCCGTGCCTGGAGgGC     80

TdHsp26-A3Ch    CGCGAGGCCGGCGCCGCTCTCGACCGGCGGGAGAACCCGC    120
  Hsp26-A3      CGCGAGGCCGGCGCCGCTCTCGACCGGCGGGAGAACCCGC    120
TdHsp26-A2Ch    CGCGAGGCCaGCGCCGCTCTCGACCGGCGGGAGAACCCGC    120
TdHsp26-A1Ch    CGCGAGGCCGGCcCCGgTCTgGAC...CGGGAGAACCCGC    114
TdHsp26-B1Ch    CGCGAGGCCaGCGCCGCTCTCGACCGGCGGGAGAACCCGC    120

TdHsp26-A3Ch    CCGCTCTTCGTGGCCTCCGCGGCGCAGGAGAATAGGGACA    160
  Hsp26-A3      CCGCTCTTCGTGGCCTCCGCGGCGCAGGAGAATAGGGACA    160
TdHsp26-A2Ch    CCGCTCTcCGTGGCCTCtGCGGCGCAGGAGAAcAGGGACA    160
TdHsp26-A1Ch    CCGCTCTcCGTGGCCTCCGCGGCGCAGGAGAAcAGaGACA    154
TdHsp26-B1Ch    CCGCTCTcCGTGGCCTCCGCGGCGCAGGAGAAcAGGGACA    160

TdHsp26-A3Ch    ACTCCGTCGACGTCCAAGTCAGCCAGGCCCAGAACGCCGG    200
  Hsp26-A3      ACTCCGTCGACGTCCAAGTCAGCCAGGCCCAGAACGCCGG    200
TdHsp26-A2Ch    ACTCCGTCGACGTCCAtGTCAG.CAGG..CAGgACGgCGG    197
TdHsp26-A1Ch    ACTCCGTCGACGTCCAAGTCAGCCAGGCCCAGAACGCtGG    194
TdHsp26-B1Ch    ACTCCGTCGACGTCCAAGTCAGCCAGGCCCAGAACGCCGG    200

TdHsp26-A3Ch    CAACCAGCAGGGCAATGCAGTCCAGCGCCGCCCTCGTCGC    240
  Hsp26-A3      CAACCAGCAGGGCAATGCAGTCCAGCGCCGCCCTCGTCGC    240
TdHsp26-A2Ch    CAACCAGCAGGGCAATGCAGTCCAGCGtCGCCCgCGcCaC    237
TdHsp26-A1Ch    aAACCAGCAGGGCAATGCAGTCCAGCGCCGCCCTCGTCGC    234
TdHsp26-B1Ch    aAACCAGCAGGGCAAcGCAGTCCAGCGCCGCCCTCGTCGC    240

TdHsp26-A3Ch    GCTGGATTTGACATCTCCCCGTTCGGTAAGTCCTCTGTTC    280
  Hsp26-A3      GCTGGATTTGACATCTCCCCGTTCGGTAAGTCCTCTGTTC    280
TdHsp26-A2Ch    GCTGGcTTcGACATCTCCCCGTTCGGTAAGTCCTCTGTTC    277
TdHsp26-A1Ch    GCTGGATTTGACATCTCCCCGTTCGGTAAGTCCTC.GTTC    273
TdHsp26-B1Ch    GCTGGATTTGACATCTCCCCGTTCGGTAAGTCCTCTGTTC    280


TdHsp26-A3Ch    CGCAGTGTCTTTCAACGAGAGAAATTTGTTCGA.GCGTGG    319
  Hsp26-A3      CGCAGTGTCTTTCAACGAGAGAAATTTGTTCGA.GCGTGG    319
TdHsp26-A2Ch    CGatGTtctTcaacgaGAGAGAAtTTTGTggcA.......    310
TdHsp26-A1Ch    CtgAtgtTCTTTtAcacg..GAAtTTTGTTgcAgGtGTaG    311
TdHsp26-B1Ch    CGCAGTGcCTTTCAAaGAGAGAAATTTGTTCcA.GtGTGa    319

TdHsp26-A3Ch    GAGAAGCAGAGCCTGACGTACGTTGTTTAGGCTAACGTAT    359
  Hsp26-A3      GAGAAGCAGAGCCTGACGTACGTTGTTTAGGCTAACGTAT    359
TdHsp26-A2Ch    .....GCAGAGGcTGACGTACGTgGTTTAcGCcAACaTAT    345
```

Supplementary Figure S2. Continue

```
TdHsp26-A1Ch    GAGAAGCAGAGCaTGACG......GTTTActtcAACGaAT   345
TdHsp26-B1Ch    GAGAAGCAGAGCCTGACGTACGTcGTTTAcGCTAACGTAT   359

TdHsp26-A3Ch    GCAGGGCTAGTGGACCCGATGTCGCCGATGAGGACGATGC   399
  Hsp26-A3      GCAGGGCTAGTGGACCCGATGTCGCCGATGAGGACGATGC   399
TdHsp26-A2Ch    GCAGGGCTgGTGGACCCGATGTCGCCGATGAGGACGATGC   385
TdHsp26-A1Ch    GCAGGGCTAGTGGACCCGATGTCGCCGATGAGGACGATGC   385
TdHsp26-B1Ch    aCAGGGCTAGTGGACCCGATGTCGCCGATGAGGACGATGC   399

TdHsp26-A3Ch    GGCAGATGCTGGACACGATGGACCGGCTGTTCGACGACGC   439
  Hsp26-A3      GGCAGATGCTGGACACGATGGACCGGCTGTTCGACGACGC   439
TdHsp26-A2Ch    GGCAGATGCTGGACACGATGGACCGGCTGTTCGACGACGC   425
TdHsp26-A1Ch    GGCAGATGCTGGACACGATGGACCGGCTGTTCGACGACGC   425
TdHsp26-B1Ch    GGCAGATGCTtGACACGATGGACCGGCTGTTCGACGACGC   439

TdHsp26-A3Ch    CGTGGGGTTCCCCACGGCGCGTCGCTCGCCAGCGGCGGCG   479
  Hsp26-A3      CGTGGGGTTCCCCACGGCGCGTCGCTCGCCAGCGGCGGCG   479
TdHsp26-A2Ch    CGTGGGGTTCCCCACGGCGCGgCGCTCGCCAGCGGCGGCG   465
TdHsp26-A1Ch    CGTGGGGTTCCCCACGGCGCGgCGCTCGCCgGCGGCGGCG   465
TdHsp26-B1Ch    CGTGGGGTTCCCCACGGCGCGgCGCTCGCtgGCGGCGGCG   479

TdHsp26-A3Ch    AGCGAGACGCCGTGGATGCCGTGGGACATCATGGAGGACG   519
  Hsp26-A3      AGCGAGACGCCGTGGATGCCGTGGGACATCATGGAGGACG   519
TdHsp26-A2Ch    AGCGAGACGCCGcGGATGCCGTGGGACATCATGGAGGACG   505
TdHsp26-A1Ch    AGCGAGACGCCGcGGATGCCGTGGGACATtATGGAGGACG   505
TdHsp26-B1Ch    AGCGAGAtGCCGcGGATGCCGTGGGACATCATGGAGGACG   519

TdHsp26-A3Ch    AGAAGGAGGTGAAGATGCGGTTTGACATGCCTGGGCTGTC   559
  Hsp26-A3      AGAAGGAGGTGAAGATGCGGTTTGACATGCCTGGGCTGTC   559
TdHsp26-A2Ch    AcAAGGAGGTGAAGATGCGGTTcGACATGCCcGGGCTGTC   545
TdHsp26-A1Ch    AGAAGGAGGTGAAGATGCGGTTTGACATGCCTGGGCTGTC   545
TdHsp26-B1Ch    AcAAGGAGGTGAAGATGCGGTTcGACATGCCcGGGCTGTC   559

TdHsp26-A3Ch    GCGGGAGGAGGTGAGGGTGATGGTGGAGGACGACGCGCTG   599
  Hsp26-A3      GCGGGAGGAGGTGAGGGTGATGGTGGAGGACGACGCGCTG   599
TdHsp26-A2Ch    GCGGGAGGAGGTGAaGGTGATGGTGGAGGgCGACGCGCTc   585
TdHsp26-A1Ch    GCGGGAGGAGGTGAGGGTGATGGTGGAGGACGACGCGCTG   585
TdHsp26-B1Ch    GCGGGAGGAGGTGAaGGTGATGGTGGAGGgCGACGCGCTc   599

TdHsp26-A3Ch    GTCATCCGCGGCGAGCACGGTTAG................   623
  Hsp26-A3      GTCATCCGCGGCGAGCACGGTTAGactatgtatagcttct   639
TdHsp26-A2Ch    GTCATCCGCGGCGAGCAC......................   603
TdHsp26-A1Ch    GTCATCCGCGGCGAGCAC......................   603
TdHsp26-B1Ch    GTCATCCGCGGCGAGCAC......................   617

TdHsp26-A3Ch    ........................................   623
  Hsp26-A3      gtacctatgtacgtatatggtacatattgtaacacaacca   679
TdHsp26-A2Ch    ........................................   603
TdHsp26-A1Ch    ........................................   603
TdHsp26-B1Ch    ........................................   617

TdHsp26-A3Ch    ........................................   623
  Hsp26-A3      ttatatataatgagataagccacctctagaaggtacatat   719
TdHsp26-A2Ch    ........................................   603
TdHsp26-A1Ch    ........................................   603
TdHsp26-B1Ch    ........................................   617
```

Supplementary Figure S2. Continue

```
TdHsp26-A3Ch    ........................................   623
  Hsp26-A3      tgtaacacaatcattatatataatgagataagctacccct   759
TdHsp26-A2Ch    ........................................   603
TdHsp26-A1Ch    ........................................   603
TdHsp26-B1Ch    ........................................   617

TdHsp26-A3Ch    ........................................   623
  Hsp26-A3      agagggttgtgctggttccccaaaacttattgtcttacat   799
TdHsp26-A2Ch    ........................................   603
TdHsp26-A1Ch    ........................................   603
TdHsp26-B1Ch    ........................................   617

TdHsp26-A3Ch    ........................................   623
  Hsp26-A3      ggtatcacgctaggttacgatcgcttccgcttctaaaccc   839
TdHsp26-A2Ch    ........................................   603
TdHsp26-A1Ch    ........................................   603
TdHsp26-B1Ch    ........................................   617

TdHsp26-A3Ch    ........................................   623
  Hsp26-A3      taatacccgcaccgccgtcgcagccgccgccgccttcact   879
TdHsp26-A2Ch    ........................................   603
TdHsp26-A1Ch    ........................................   603
TdHsp26-B1Ch    ........................................   617

TdHsp26-A3Ch    ........................................   623
  Hsp26-A3      gccgctgccgcgccaccgatcgcgccgccgctatgtcgag   919
TdHsp26-A2Ch    ........................................   603
TdHsp26-A1Ch    ........................................   603
TdHsp26-B1Ch    ........................................   617

TdHsp26-A3Ch    ........................................   623
  Hsp26-A3      cgccgccaccaccagttccactgctgcgggcttcctcccg   959
TdHsp26-A2Ch    ........................................   603
TdHsp26-A1Ch    ........................................   603
TdHsp26-B1Ch    ........................................   617

TdHsp26-A3Ch    ........................................   623
  Hsp26-A3      gcctctcttgcggctctgctcaacctcccgctcgatgccg   999
TdHsp26-A2Ch    ........................................   603
TdHsp26-A1Ch    ........................................   603
TdHsp26-B1Ch    ........................................   617

TdHsp26-A3Ch    ........................................   623
  Hsp26-A3      tctctgttccggctccgatcgggacaaggagcatcgactc  1039
TdHsp26-A2Ch    ........................................   603
TdHsp26-A1Ch    ........................................   603
TdHsp26-B1Ch    ........................................   617

TdHsp26-A3Ch    ........................................   623
  Hsp26-A3      cgtcttctccacgccgctggcgccctcgcttgggcgtgac  1079
TdHsp26-A2Ch    ........................................   603
TdHsp26-A1Ch    ........................................   603
TdHsp26-B1Ch    ........................................   617

TdHsp26-A3Ch    ........................................   623
  Hsp26-A3      ctcgtggtccacaccgcggcgccgccgtccgctgcggact  1119
TdHsp26-A2Ch    ........................................   603
TdHsp26-A1Ch    ........................................   603
TdHsp26-B1Ch    ........................................   617
```

Supplementary Figure S2. Continue

```
TdHsp26-A3Ch     ........................................   623
  Hsp26-A3       ccgcaggcgtcgtcccgctgctcctgccgcaagcggatca  1159
TdHsp26-A2Ch     ........................................   603
TdHsp26-A1Ch     ........................................   603
TdHsp26-B1Ch     ........................................   617

TdHsp26-A3Ch     ........................................   623
  Hsp26-A3       cactgccccgctggcggggttgacggcgttcgccccgacc  1199
TdHsp26-A2Ch     ........................................   603
TdHsp26-A1Ch     ........................................   603
TdHsp26-B1Ch     ........................................   617

TdHsp26-A3Ch     ........................................   623
  Hsp26-A3       gcgggccttgcggcgtctgcaccggccgcgagctttgcgg  1239
TdHsp26-A2Ch     ........................................   603
TdHsp26-A1Ch     ........................................   603
TdHsp26-B1Ch     ........................................   617

TdHsp26-A3Ch     ........................................   623
  Hsp26-A3       cgcccgccctggctgcggtcccgcctcctcccgcatcggc  1279
TdHsp26-A2Ch     ........................................   603
TdHsp26-A1Ch     ........................................   603
TdHsp26-B1Ch     ........................................   617

TdHsp26-A3Ch     ........................................   623
  Hsp26-A3       ttcggtgtctccggctgcctccatggtgtttgcaccccag  1319
TdHsp26-A2Ch     ........................................   603
TdHsp26-A1Ch     ........................................   603
TdHsp26-B1Ch     ........................................   617

TdHsp26-A3Ch     ........................................   623
  Hsp26-A3       gcagcctcctcgatgggattgtcttcgccgccgccgtttc  1359
TdHsp26-A2Ch     ........................................   603
TdHsp26-A1Ch     ........................................   603
TdHsp26-B1Ch     ........................................   617

TdHsp26-A3Ch     ........................................   623
  Hsp26-A3       acttcggtcatttcatcaccatcaagctctccgccgacaa  1399
TdHsp26-A2Ch     ........................................   603
TdHsp26-A1Ch     ........................................   603
TdHsp26-B1Ch     ........................................   617

TdHsp26-A3Ch     ........................................   623
  Hsp26-A3       ctacatcttctggcgtgcgcaggttctcccgctcttgggg  1439
TdHsp26-A2Ch     ........................................   603
TdHsp26-A1Ch     ........................................   603
TdHsp26-B1Ch     ........................................   617

TdHsp26-A3Ch     ........................................   623
  Hsp26-A3       agtcactacctgctaggctacatcgacggatcgcttccct  1479
TdHsp26-A2Ch     ........................................   603
TdHsp26-A1Ch     ........................................   603
TdHsp26-B1Ch     ........................................   617

TdHsp26-A3Ch     ........................................   623
  Hsp26-A3       gcccacccgcgttggtagacagcgtgcatggtccggtcta  1519
TdHsp26-A2Ch     ........................................   603
TdHsp26-A1Ch     ........................................   603
```

Supplementary Figure S2. Continue

```
TdHsp26-B1Ch     ........................................   617
TdHsp26-A3Ch     ........................................   623
  Hsp26-A3       caatccggcccatcgcgtctggacggggcaggaccaggcg  1559
TdHsp26-A2Ch     ........................................   603
TdHsp26-A1Ch     ........................................   603
TdHsp26-B1Ch     ........................................   617


TdHsp26-A3Ch     ........................................   623
  Hsp26-A3       aacctctcctccatccaggggtcgctctcgccggcagttg  1599
TdHsp26-A2Ch     ........................................   603
TdHsp26-A1Ch     ........................................   603
TdHsp26-B1Ch     ........................................   617

TdHsp26-A3Ch     ........................................           623
  Hsp26-A3       tcggccttgttgtcttcgcg(3'828N)ctttaccgctgccacagttt    5467
TdHsp26-A2Ch     ........................................           603
TdHsp26-A1Ch     ........................................           603
TdHsp26-B1Ch     ........................................           617

TdHsp26-A3Ch     ........................................   623
  Hsp26-A3       gaggcttgtaggcgcaatcttacccttctcagttctttag  5507
TdHsp26-A2Ch     ........................................   603
TdHsp26-A1Ch     ........................................   603
TdHsp26-B1Ch     ........................................   617

TdHsp26-A3Ch     ........................................   623
  Hsp26-A3       aaagtggctaagattgagggagggtgttagactatgtata  5547
TdHsp26-A2Ch     ........................................   603
TdHsp26-A1Ch     ........................................   603
TdHsp26-B1Ch     ........................................   617

TdHsp26-A3Ch     ........................................   623
  Hsp26-A3       gcttctgtacctatgtatgtatatggtacatattgtaaca  5587
TdHsp26-A2Ch     ........................................   603
TdHsp26-A1Ch     ........................................   603
TdHsp26-B1Ch     ........................................   617

TdHsp26-A3Ch     ........................................   623
  Hsp26-A3       caaccattatatataatgagataagccacccctagagggt  5627
TdHsp26-A2Ch     ........................................   603
TdHsp26-A1Ch     ........................................   603
TdHsp26-B1Ch     ........................................   617

TdHsp26-A3Ch     ........................................   623
  Hsp26-A3       tgtgctggttccccaaaacatattgtcttacaagcacaag  5667
TdHsp26-A2Ch     .....................................aag   606
TdHsp26-A1Ch     .....................................aag   606
TdHsp26-B1Ch     .....................................aag   620

TdHsp26-A3Ch     ..................................................    623
  Hsp26-A3       aaggaggccggcgaagggcagggc.........gaaggcggcgacgggt   5707
TdHsp26-A2Ch     gaggaggccggcgaagggcagggc.........gaaggcggcgacgggt    646
TdHsp26-A1Ch     aaggaggccggcgaagggcagggc.........gaaggcggcgacgggt    646
TdHsp26-B1Ch     aaggaggccggcgaaggccagggcgaagcagcggaaggcggcgacgggt    669

TdHsp26-A3Ch     ........................................   623
  Hsp26-A3       ggtggaaggagcgcagcgtgagctcctacgacatgtgcct  5747
```

Supplementary Figure S2. Continue

```
TdHsp26-A2Ch      ggtggaaggagcgcagcgtgagctcctacgacatgcgcct    686
TdHsp26-A1Ch      ggtggaaggagcgcagcgtgagctcctacgacatgcgact    686
TdHsp26-B1Ch      ggtggaaggagcgcagcgtgagctcctacgacatgcgcct    709

TdHsp26-A3Ch      ........................................    623
  Hsp26-A3        tgctctgccggacgagtgcgacaagagccaggtgcgggcc   5787
TdHsp26-A2Ch      ggctctgccggacgagtgcgacaagagccaggtgcgggcc    726
TdHsp26-A1Ch      ggctctgccggacgagtgcgacaagagccaggtgcgcgcc    726
TdHsp26-B1Ch      ggctctgccggacgagtgcgacaagagtcaggtgcgcgcc    749


TdHsp26-A3Ch     ........................................    623
  Hsp26-A3       gagctcaagaacggcgtgctgctcgtgttcgtgcccaaga   5827
TdHsp26-A2Ch     gagctcaagaacggcgtgctgctcgtgtccgtgcccaaga    766
TdHsp26-A1Ch     gagctcaagaacggcgtgctgctcgtgtccgtgcccaaga    766
TdHsp26-B1Ch     gagctcaagaacggcgtgctgctcgtgtccgtgcccaaga    789

TdHsp26-A3Ch     ........................................    623
  Hsp26-A3       gggagaccgagcgcaaggtcatcgacgtgcaggtccagtg   5867
TdHsp26-A2Ch     gggagaccgagcgcaaggtcatcgacgtgcaggtccagtg    806
TdHsp26-A1Ch     gggagaccgagcgcaaggtcatcgacgtgcaggtccagtg    806
TdHsp26-B1Ch     gggagaccgagcgcaaggtcatcgacgtgaaggtccagtg    829

TdHsp26-A3Ch     ........................................    623
  Hsp26-A3       a.......................................   5868
TdHsp26-A2Ch     a.......................................    807
TdHsp26-A1Ch     a.......................................    807
TdHsp26-B1Ch     a.......................................    830
```
