# Supplementary material for: Heat in Wheat: Exploit Reverse Genetic Techniques to Discover New Alleles Within the Triticum durum sHsp26 Family
Source: Front Plant Sci. 2018 Sep 19;9:1337. doi: 10.3389/fpls.2018.01337 (PMC6156267; doi:10.3389/fpls.2018.01337)
Supplement: Supplementary file 8 [file Image_3.PDF]

Supplementary Material

Heat in wheat: exploit reverse genetic techniques to discover new alleles within the *Triticum durum* sHsp26 family

Alessia Comastri, Michela Janni*, James Simmonds [4], Cristobal Uauy[4], Domenico Pignone[2], Henry T. Nguyen[5], Nelson Marmiroli[1].

*** Correspondence:** Corresponding Author: michela.janni@ibbr.cnr.it

Supplementary Figure S3 Multiple alignment of chloroplast-localized sHSP26 sequences harboured by Triticum spp. and other plant species. TdHSP26-A1Ch (CZQ76680), TdHSP26-A1Kr (CZQ76681), TdHSP26.5 (CAI96515), TaHSP26.6m (AAC96317), TaHSP26.6B (CAA47745), TdHSP26-B1Ch (CZQ76686), TaHSP26.6g (AAAC96315), TdHSP26-A2Ch (CZQ76682), TdHSP26-A2Kr (CZQ76683), TdHSP26.4 (CAI96512), HSP26-A3, TaHSP26.6i (AAC96316), BdsHSP (XP_003558381), HvHSP26 (AAB28590), ZmHSP26 (AAA33477), ObHSP26.7 (XP_006649731), LeHSP26.1 (AAB49626), AtHSP21 (CAB38279), VvHSP25(CAO48583), CaHSP26.13 (AFZ94855), PsHSP26.2 (CAA30167).
The α-helix and β-sheets predicted by the PROMALS3D structural alignment program (prodata.swmed.edu/promals3d/promals3d.php; Pei et al. 2008) are colored red and blue respectively. The transit peptide cleavage site is arrowed. The conserved N terminal MrD, ACD β-sheets and CRI and CRII (Scharf et al. 2001; Waters 2013) are shown.

```
                                  Transit peptide
TdHSP26-A1Ch    1   MAAANAPFAL-SRLSPAARLPFR-------AWRAARPAPV---WT-GRTR----PLSVASAAQE--NRDN   52
TdHSP26-A1Kr    1   MAAANAPFAL-SRLSPAARLPFR-------AWRAARPAPV---WT-GRTR----PLSVASAAQE--NRDN   52
TdHSP26.5       1   MAAANAPFAL-SRLSPAARLPFR-------AWRAARPAPV---WT-GRTR----PLSVASAAQE--NRDN   52
TaHSP26.6m      1   MAAANAPFAL-SRLSPAARLPFR-------AWRAARPAPV---WT-GRTR----PLSVASAAQE--NRDN   52
TaHSP26.6B      1   MAAANAPFAL-SRLSPAARLPFR-------AWRAARPAPV---WT-GRTR----PLSVASAAQE--NRDN   52
TdHSP26-B1Ch    1   MADANAPFALVSRLSPAARLPIR-------AWRAARPAPL--STG-GRTR----PLSVASAAQE--NRDN   54
TaHSP26.6g      1   MADANAPFALVSRLSPAARLPIR-------AWRAARPAPL--STG-GRTR----PLSVASAAQE--NRDN   54
TdHSP26-A2Ch    1   MAAANAPFAIVSRLSPAARLPIR-------AWRAARPAPL--STG-GRTR----PLSVASAAQE--NRDN   54
TdHSP26-A2Kr    1   MAAANAPFAIVSRLSPAARLPIR-------AWRAARPAPL—-STG-GRTR----PLSVASAAQE--NRDN   54
TdHSP26.4       1   MAAANAPFAIVSRLSPAARLPIR-------AWRAARPAPL--STG-GRTR----PLSVASAAQE--NRDN   54
HSP26-A3        1   MAAANAPFALVSRLSPAARLPIR-------AWRAARPAPL--STG-GRTR----PLFVASAAQE--NRDN   54
TaHSP26.6i      1   MAAANAPFALVSRLSPAARLPIR-------AWRAARPAPL--STG-GRTR----PLSVASAAQE--NRDN   54
BdsHSP          1   MAAAAAPFGLVSRLSPAARLPIR-------AWRAARPAPLGITST-GRTR----PLSVASAAQE--NRDI   56
HvHSP26         1   MAAATAPFALVSRLSQAARLPIR-------AWRAARPAPL---WT-GRTR----PLSVASAAQE--DRDN   53
ZmHSP26         1   M--AAAPFAIAGRLSPVARLPVR-------AWRPAH----GFASS-GRAR----SLAVASAAQE--NRDN   50
ObHSP26.7       1   M---AAPFALVSRVSPAARLPIR------AAWRNARPT-VGLPSS-GRVR----QLAVASAAQE--NRDN   53
LeHSP26.1       1   M------AYTSLTSSPLVSNVSVGGTSKINNNKVSAPCSVFVPSM-RRPT----TRLVARATGD--NKDT   57
AtHSP21         1   M-ASTLSFAASALCSPLAPSPSV-------SSKSATPFSVSFP---RKIP----SRIRAQD-----QREN   50
VvHSP25         1   M---ASLKAFPFSSSPLATHKPS-----LSKGVAGAPCSAFFPSS-RHGGRSRLALVRAEATGE--NKDA   59
CaHSP26.13      1   M-ASKGITCSASSAAPLVSSRTT-----SSKLVGLGPCSVTFPAP-KKPSLGSRLVVRAQQGGE-QNKEG   62
PsHSP26.2       1   M---AQSVSLSTIASPILSQKPG------SSVKSTPPCMASFPLR-RQLPRLGLRNVRAQAGGDGDNKDN   60
                              __β___α_   α_      __α__            ______α____

                                                                Met-rich region (MrD)
TdHSP26-A1Ch   53   S--VDVQVSQAQNAGN-QQGNAVQRRP-RRA-GFDI--SPFGLVDPMSPMRTMRQMLDTMDRLFDD-AVG  114
TdHSP26-A1Kr   53   S--VDVQVSQAQNAGN-QQGNAVERRP-RRA-GFDI--SPFGLVDPMSPMRTMRQMLDTMDRLFDD-AVG  114
TdHSP26.5      53   S--VDVQVSQAQNAGN-QQGNAVERRP-RRA-GFDI--SPFGLVDPMSPMRTMRQMLDTMDRLFDD-AVG  114
TaHSP26.6m     53   S--VDVQVSQAQNAGN-QQGNAVQRRP-RRA-GFDI--SPFGLVDPMSPMRTMRQMLDTMDRLFDD-AVG  114
TaHSP26.6B     53   S--VDVQVSQAQNAGN-QQGNAVQRRP-RRA-GFDI--SPFGLVDPMSPMRTMRQMLDTMDRLFDD-AVG  114
TdHSP26-B1Ch   55   S--VDVQVSQAQNAGN-QQGNAVQRRP-RRA-GFDI--SPFGLVDPMSPMRTMRQMLDTMDRLFDD-AVG  116
TaHSP26.6g     55   S--VDVQVSQAQNAGN-QQGNAVQRRP-RRA-GFDI--SPFGLVDPMSPMRTMRQMLDTMDRLFDD-AVG  116
TdHSP26-A2Ch   55   S--VDVHVSR-QDGGN-QQGNAVQRRP-RHA-GFDI--SPFGLVDPMSPMRTMRQMLDTMDRLFDD-AVG  115
TdHSP26-A2Kr   55   S--VDVHVSR-QDGGN-QQGNAVQRRP-RHA-GFDI--SPFGLVDPMSPMRTMRQMLDTMDRLFDD-AVG  115
TdHSP26.4      55   S--VDVHVSR-QDGGN-QQGNAVQRRP-RHA-GFDI--SPFGLVDPMSPMRTMRQMLDTMDRLFDD-AVG  115
HSP26-A3       55   S--VDVQVSQAQNAGN-QQGNAVQRRP-RRA-GFDI--SPFGLVDPMSPMRTMRQMLDTMDRLFDD-AVG  116
TaHSP26.6i     55   S--VDVQVSQAQNAGN-QQGNAVQRRP-RRA-GFDI--SPFGLVDPMSPMRTMRQMLDTMDRLFDD-AVG  116
BdsHSP         57   SS-LDVQVS--QNGGN-QQGNAVQRRP-RRA-GFDV--SPFGLVDPMSPMRTMRQMLDTMDRLFDD-TVG  117
HvHSP26        54   S--VDVQVSQARNAGN-QQGNAVQRRP-RRA-GFDI--SPFGLVDPMSPMRTMKQMSDTMDRLFDD-AVG  115
ZmHSP26        51   S--VDVQVS--QNGGNRQQGNAVQRRP-RRATALDISPSPFGLVDPMSPMRTMRQMLDTMDRLFDD-AVG  114
ObHSP26.7      54   TA-VDVHVN--QDGGN-QQGNAVQRRP-RRS-AFDI--SPFGLVDPFSPMRTMRQMLDTMDRMFDDVAVG  115
LeHSP26.1      58   S--VDVHHSSAQGGN--NQGTAVERRP-TRM-ALDV--SPFGVLDPMSPMRTMRQMIDTMDRLFED-TM-  117
AtHSP21        51   S--IDVV---QQGQQKGNQGSSVEKRPQQRL-TMDV--SPFGLLDPLSPMRTMRQMLDTMDRMFED-TMP  111
VvHSP25        60   S--LDVQV---HQGN--KGATAVERQP-RRL-ALDI--SPFGLLDPFSPMRTMRQMMDAMDRMFEE-TVA  117
CaHSP26.13     63   SHHVDVQV---QNTNR-QQSSAVERRP-RRL-AVDM--SPFGLIDSLSPMRSMRQMLDTMDRLFED-TMT  123
PsHSP26.2      61   S--VEV-----HRVNKDDQGTAVERKP-RRS-SIDI--SPFGLLDPWSPMRSMRQMLDTMDRIFED-AIT  118
                      ___β____            __β_                   ________α_________
```

Supplementary Figure S3. Continue

```
                                                          Consensus Region II
                                                   α-crystallin domain (ACD)
TdHSP26-A1Ch  115  FPTARRSPAAA-SETPRMPWDIMEDEKEVKMRFDMPGLSREEVRVMVEDDALVIRGEHKKEAGEG---QG  180
TdHSP26-A1Kr  115  FPTARRSPAAA-SETPRMPWDIMEDEKEVKMRFDMPGLSREEVRVMVEDDALVIRGEHKKEAGEG---QG  180
TdHSP26.5     115  FPTARRSPAAA-SETPRMPWDIMEDEKEVKMRFDMPGLSREEVRVMVEDDALVIRGEHKKEAGEG---QG  180
TaHSP26.6m    115  FPTARRSPAAA-SETPRMPWDIMEDEKEVKMRFDMPGLSREEVRVMVEDDALVIRGEHKKEAGEG---QG  180
TaHSP26.6B    115  FPTA-RSPARR-AKTPRMPWDIMEDEKEVKMRFDMPGLSREEVRVMVEDDALVIRGEHKKEAGEG---QG  179
TdHSP26-B1Ch  117  FPTARRSLAAA-SEMPRMPWDIMEDDKEVKMRFDMPGLSREEVKVMVEGDALVIRGEHKKEAGEGQGEAA  185
TaHSP26.6g    117  FPTARRSLAAA-SEMPRMPWDIMEDDKEVKMRFDMPGLSREEVKVMVEGDALVIRGEHKKEAGEGQGEAA  185
TdHSP26-A2Ch  116  FPTARRSPAAA-SETPRMPWDIMEDDKEVKMRFDMPGLSREEVKVMVEGDALVIRGEHKEEAGEGQG---  181
TdHSP26-A2Kr  116  FPTARRSPAAA-SETPRMPWDIMEDDKEVKMRFDMPGLSREEVKVMVEGDALVIRGEHKKEAGEGQG---  181
TdHSP26.4     116  FPTARRSPAAA-SETPRMPWDIMEDDKEVKMRFDMPGLSREEVKVMVEGDALVIRGEHKKEAGEGQG---  181
HSP26-A3      117  FPTARRSPAAA-SETPWMPWDIMEDEKEVKMRFDMPGLSREEVRVMVEDDALVIRGEHKKEAGEGQG---  182
TaHSP26.6i    117  FPTARRSPAAA-SETPRMPWDIMEDEKEVKMRFDMPGLSREEVRVMVEDDALVIRGEHKKEAGEGQG---  182
BdsHSP        118  FPTARGRSPAA-SET-RMPWDIMEDDKEVKMRFDMPGLSREEVKVSVEDDALVIRGEHRKEAGED----A  181
HvHSP26       116  FPTARRSPAAAAGEMPRMPWDIMEDDKEVKMRFDMPGLSREEVKVMVEGDALVIRGEHKKEAGEGQGEAA  185
ZmHSP26       115  FPMGTRRSPATTGDV-RLPWDIVEDEKEVKMRIDMPGLARDEVKVMVEDDTLVIRGEHKKEEGAE-GGSG  182
ObHSP26.7     116  FPAAPRRSPVT-GEV-RMPWDVMEDDKEVKMRFDMPGLSREEVKVMVEDDALVIRGEHKKEEGEG---AE  180
LeHSP26.1     118  IPGRNR-ASGT-GEI-RTPWDIHDDENEIKMRFDMPGLSKEDVKVSVENDMLVIKGEHKKEED-------  177
AtHSP21       112  VSGRNRGGSGV-SEI-RAPWDIKEEEHEIKMRFDMPGLSKEDVKISVEDNVLVIKGEQKKED--------  171
VvHSP25       118  FPGS--------AEV-RSPWDIVDDENEIKMRFDMPGLSKEDVKVSVEDDLLVIKGEQKKEEG-------  171
CaHSP26.13    124  VPTRM-------GEM-RAPWDIMEDENEYKMRFDMPGLDKGDVKVSVEDNMLVIKGERKKEEG-------  178
PsHSP26.2     119  IPGRNIGG----GEI-RVPWEIKDEEHEIRMRFDMPGVSKEDVKVSVEDDVLVIKSDHREENG-------  176
                                     __β2__   ___β3__   _α___β4__   ___β5___
```

```
                                       Consensus Region I
                         α-crystallin domain (ACD)
TdHSP26-A1Ch  181  EGGDGWWK-ERSVSSYDMRLALPDECDKSQVRAELKNGVLLVSVPKRETERKVIDVQVQ  238
TdHSP26-A1Kr  181  EGGDGWWK-ERSVSSYGMRLALPDECDKSQVRAELKNGVLLVSVPKRETERKVIDVQVQ  238
TdHSP26.5     181  EGGDGWWK-ERSVSSYGMRLALPDECDKSQVRAELKNGVLLVSVPKRETERKVIDVQVQ  238
TaHSP26.6m    181  EGGDGWWK-ERSLSSYDMRLALPDECDKSQVRAELKNGVLLVSVPKRETERKVIDVQVQ  238
TaHSP26.6B    180  EGGDGWWK-ERSLSSYDMRLALPDECDKSQVRAELKNGVLLVSVPKRETERKVIDVQVQ  237
TdHSP26-B1Ch  186  EGGDGWWK-ERSVSSYDMRLALPDECDKSQVRAELKNGVLLVSVPKRETERKVIDVKVQ  243
TaHSP26.6g    186  EGGDGWWK-ERSVSSYDMRLALPDECDKSQVRAELKNGVLLVSVPKRETERKVIDVKVQ  243
TdHSP26-A2Ch  182  EGGDGWWK-ERSVSSYDMRLALPDECDKSQVRAELKNGVLLVSVPKRETERKVIDVQVQ  239
TdHSP26-A2Kr  182  EGGDGWWK-ERSVSSYDMRLALPDECDKSQVRAELKNGVLLVSVPKRETERKVIDVQVQ  239
TdHSP26.4     182  EGGDGWWK-ERSVSSYDMRLALPDECDKSQVRAELKNGVLLVSVPKRETERKVIDVQVQ  239
HSP26-A3      183  EGGDGWWK-ERSVSSYDMCLALPDECDKSQVRAELKNGVLLVFVPKRETERKVIDVQVQ  240
TaHSP26.6i    183  EGGDGWWK-ERSVSSYDMRLALPDECDKSQVRAELKNGVLLVSVPKRETERKVIDVQVQ  240
BdsHSP        182  EGGDGWWK-ERSVSSYDMRLALPDTCDKSQVRAELKNGVLLVTVPKTETEHKVINVQVQ  239
HvHSP26       186  GGGDGWWK-ERSVSSYDMRLALPDECDKSQVRAELKNGVLLVSVPKRETERKVIDVQVQ  243
ZmHSP26       183  GDGDGWWK-QRSVSSYDMRLALPDECDKSKVRAELKNGVLLVTVPKTEVERKVIDVQVQ  240
ObHSP26.7     181  GAGDGWWK-ERSVSSYDMRLPLPDGCDKSKVRAELKNGVLLVTVPKTEVERKVIDVQVQ  238
LeHSP26.1     178  --GRDKHSWGRNYSSYDTRLSLPDNVVKDKIKAELKNGVLFISIPKTEVEKKVIDVQIN  234
AtHSP21       172  --SDDSWS-GRSVSSYGTRLQLPDNCEKDKIKAELKNGVLFITIPKTKVERKVIDVQIQ  227
VvHSP25       172  --EKDSWS-GSGFSSYSTRLQLPDNCEKDKIKAELKNGVLSISIPKTKVERKVIDVQIQ  227
CaHSP26.13    179  --GDDAWS-KRSYSSYDTRLQLPDNCELDKIKAELKNGVLNISIPKPKVERKVIDVQIQ  234
PsHSP26.2     177  --GEDCWS-RKSYSCYDTRLKLPDNCEKEKVKAELKDGVLYITIPKTKIERTVIDVQIQ  232
                         __β6___β7_____   _α___β8__   ____β9__   _β10_
```
